# Molecular Phylodynamic Analysis Indicates Lineage Displacement Occurred in Chinese Rabies Epidemics between 1949 to 2010

**DOI:** 10.1371/journal.pntd.0002294

**Published:** 2013-07-11

**Authors:** Xiao-Yan Tao, Qing Tang, Simon Rayner, Zhen-Yang Guo, Hao Li, Shu-Lin Lang, Cui-Ping Yin, Na Han, Wei Fang, James Adams, Miao Song, Guo-Dong Liang

**Affiliations:** 1 State Key Laboratory for Infectious Disease Prevention and Control, National Institute for Viral Disease Control and Prevention, Chinese Center for Disease Control and Prevention, Beijing, China; 2 Key Laboratory of Agricultural and Environmental Microbiology, Wuhan Institute of Virology, Chinese Academy of Sciences, Wuhan, Hubei, China; 3 Liupanshui Vocational and Technical College, Liupanshui, Guizhou, China; The Global Alliance for Rabies Control, United States of America

## Abstract

Rabies remains a serious problem in China with three epidemics since 1949 and the country in the midst of the third epidemic. Significantly, the control of each outbreak has been followed by a rapid reemergence of the disease. In 2005, the government implemented a rabies national surveillance program that included the collection and screening of almost 8,000 samples. In this work, we analyzed a Chinese dataset comprising 320 glycoprotein sequences covering 23 provinces and eight species, spanning the second and third epidemics. Specifically, we investigated whether the three epidemics are associated with a single reemerging lineage or a different lineage was responsible for each epidemic. Consistent with previous results, phylogenetic analysis identified six lineages, China I to VI. Analysis of the geographical composition of these lineages revealed they are consistent with human case data and reflect the gradual emergence of China I in the third epidemic. Initially, China I was restricted to south China and China II was dominant. However, as the epidemic began to spread into new areas, China I began to emerge, whereas China II remained confined to south China. By the latter part of the surveillance period, almost all isolates were China I and contributions from the remaining lineages were minimal. The prevalence of China II in the early stages of the third epidemic and its established presence in wildlife suggests that it too replaced a previously dominant lineage during the second epidemic. This lineage replacement may be a consequence of control programs that were dominated by dog culling efforts as the primary control method in the first two epidemics. This had the effect of reducing dominant strains to levels comparable with other localized background stains. Our results indicate the importance of effective control strategies for long term control of the disease.

## Author Summary

Since 1949, there have been three rabies epidemics in China. The country is currently in the midst of a third epidemic. After the first two epidemics were brought under control, there was a rapid reemergence of the disease. In 2005, the government implemented a national surveillance program and as part of this work, samples were collected from humans and animals and screened for rabies. Positive samples were sequenced and combined with other publicly available sequences to form a dataset that spanned almost all epidemic regions in China. A phylogenetic tree was constructed the clustering of isolates according to geographic origin and lineage was investigated. We found that most isolates were grouped into two lineages China I and China II. However, the proportion of isolates in these lineages changed over time until almost all new isolates were placed in China I, indicating it has emerged as the dominant lineage. Furthermore, the significantly higher number of China II isolates compared to remaining lineages together with its established presence in wildlife suggests that it was dominant in the second epidemic, suggesting that lineage replacement also occurred during the previous epidemic.

## Introduction

Rabies is a fatal enzootic disease caused by lyssaviruses and is distributed throughout most of the world, infecting a wide range of hosts [1]. In developed countries, where dog rabies has been effectively controlled, rabies remains epidemic in wild animals, with the *Carnivora* and *Chiroptera* orders acting as the main reservoirs [2]. However, most of the human cases are reported in the developing countries of Asia, Africa and Latin America [3]. According to WHO reports, more than 50,000 people die of rabies annually, with 56% of the fatalities occurring in Asia [2], [4]. Domestic dogs act as the main reservoir and are primarily responsible for the dissemination of the disease [5].

**Figure 1:**
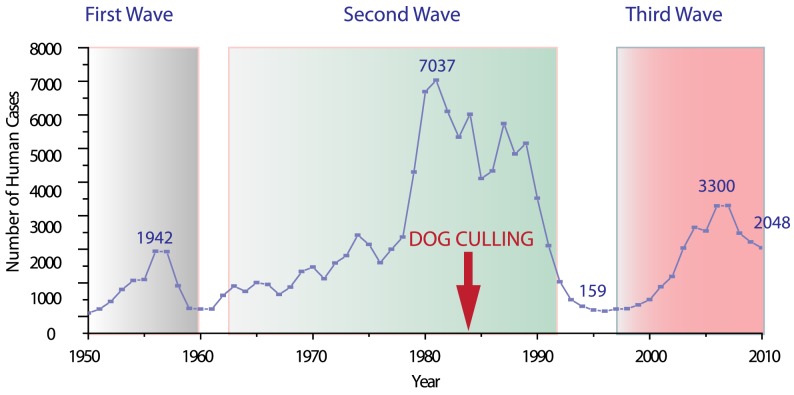
The three rabies epidemic waves in Mainland China since 1949. Y-axis shows annual number of human of rabies cases in China from 1950 to 2010. Since the foundation of the People's Republic of China, the country has experienced three rabies epidemics. The country is currently in the midst of a third epidemic that begun in 1996 (159 cases), peaked in 2007 (3300 cases) and has begun to gradually decrease since this time. Red arrow indicates the beginning of a concerted dog culling program implemented in 1984.

Rabies is widely epidemic in Asia and, after India, China has the second highest number of human rabies cases [2]. Figure 1 shows a summary of the annual number of human cases in China between 1950 and the present day. In spite of the variation in the comprehensiveness of surveillance in earlier years, the figure shows three epidemic waves over this period [6]. Moreover, the gap between successive epidemics is brief, with a rapid reemergence of cases within a few years. The first wave occurred in the 1950s, reaching a peak in 1957 (1933 cases). The second wave extended from the 1960s to the middle of 1990s and was the most serious epidemic with 4000–7000 cases reported annually for most of the 1980s, with a peak of 7037 cases occurring in 1981. China is currently in the midst of a third wave that peaked in 2007 (3300 cases). Since then, the numbers have begun to gradually decrease, although there are still around 2000 cases reported every year [6]–[8]. Thus, rabies remains a serious public health problem in China with the continuing threat of a resurgence of the disease.

The Department of Viral Encephalitis, within the National Institute for Viral Disease Control and Prevention at the Chinese Center for Disease Control and Prevention (China CDC), has been the national reference laboratory for national rabies surveillance in China since 2005. The laboratory is responsible for the identification of rabies in patients and animals at a national level using WHO approved tests, and for the subsequent characterization and analysis of positive samples. From this data, together with samples collected by other laboratories, we have been able to build a picture of the emergence of the most recent epidemic over the course of several years.

An initial investigation of the data indicated that most of the cases were located in the South of China and it was only later that cases began to occur in west and north China [7].In another paper, using surveillance data, we investigated the epidemiology of rabies in southern China [9]. We concluded that the spread of rabies viruses from high incidence regions was facilitated by the long-distance movement or trans-provincial translocation of dogs associated with human-related activities and this was likely one of the factors contributing to the rapid increase in human rabies cases in new regions [9]. Nevertheless, much remains to be learned about the epidemiology of the virus in China.

Lyssaviruses are an unsegmented, single-strand, negative strand RNA viruses of the *Rhabdoviridae* family. The lyssavirus genome is approximately 12 kb, with five genes (3′–5′) encoding the nucleoprotein (N), phosphoprotein (P), matrix protein (M), glycoprotein (G) and RNA-dependent RNA polymerase (L) [1]. As a consequence of convenience and variability, the N and G genes are the most commonly sequenced components of the genome and, based on their genetic similarity, there are currently twelve classified species within the lyssavirus genus, with an additional two putative rabies virus species Bokeloh bat lyssavirus and Ikoma lyssavirus [10]. Of these species, rabies virus (RABV) is responsible for classical rabies in terrestrial mammals globally and in bats on the American continent, as well as being associated with most rabies-related human deaths worldwide [11], [12]. Although four distinct but unclassified lyssaviruses, isolated from bats on the Asian continent, have been found in recent decades, RABV still has a major impact in Asia and remains of primary concern in China [13], [14].

Investigation of the diversity and evolution of rabies virus strains over the course of an epidemic can help in the development of strategies to combat and control viral diseases [15], [16]. In a recent paper we investigated the spatial and temporal dynamics of rabies in China based on N sequences from samples collected from 2003 to 2008 [17]. We found the epidemic was primarily defined by strains that could be divided into two major lineages which exhibited distinct population subdivision and translocation patterns. Since then, we have continued and expanded our surveillance and collected additional positive samples distributed throughout 13 provinces that were isolated from dog, human and ferret badger (*Melogale moschata*). The G protein plays a pivotal role in pathogenicity and is significantly influenced by host selection pressure [18], [19], thus this gene is a good choice for investigating the epidemiology of a rabies outbreak and hence the impact of the pathogen. Based on this, we obtained the G sequences for all new positive specimens isolated in China. While there have been several reports on the rabies epidemic in China based on the G sequence [20]–[22], these have incorporated relativity small datasets that have limited the analytical methods that could be used. As a consequence of the trial Chinese national surveillance program, as well as efforts by other rabies researchers, significantly more isolates and G sequences are now available, and the geographic distribution (23 provinces) and the range of infected hosts (8 species) has also been expanded. This more extensive dataset gives us an opportunity to reinvestigate the current epidemic to see how the characteristics have changed now the epidemic has become established across a significant portion of the country. Furthermore, given the temporal range of the samples, it allows us to compare the properties and characteristics between epidemics to try and understand how new outbreaks emerged so rapidly after a previous outbreak was brought under control.

In this work we investigated the phylodynamics of RABV in China based on the analysis of a comprehensive set of G sequences collected across the course of the current epidemic and also from the previous epidemic. We considered how the geographical dispersion of the lineages predicted from our phylogenetic analysis reflected the observed epidemiology based on surveillance data from human cases in the current epidemic. We also investigated how the prevalence of particular lineages varied within and between successive epidemics. Specifically, we considered whether successive epidemics were associated with a single lineage, or characterized by the emergence of a new lineage.

## Materials and Methods

### Ethics statement

The program for collection of human brain samples was approved by the Ethical Committee of the National Institute of Viral Disease Control and Prevention, China CDC, which is the national referral center for rabies diagnosis. Due to their medical condition, subjects were unable to provide consent once a rabies infection was suspected and so written informed consent was obtained in all cases from their relatives after death.

### Epidemiological data

Data on national human rabies cases from 1950 to 1995 were taken from the annual reports of the China CDC, formerly named the Chinese Academy of Preventive Medicine prior to 2002 (Figure 1). Data on human rabies cases from the whole of mainland China for each province and municipality (a total of 31) between 1996 and 2010 were collected from the annual reports of the China CDC. The reporting methods and how cases were determined to be associated with rabies were the same as described previously [17]. Provinces were classified as high, medium, low or very low incidence regions according to the recorded number of human cases from 1996 to 2010. In general, due to the problems of obtaining consent from relatives after a subject has died from a suspected rabies infection, human cases are not confirmed by approved by WHO laboratory testing. However, in the cases when laboratory diagnosis is performed, it was found there was a very strong correlation between positive outcome and clinical diagnosis [17]. This is primarily due to the majority of cases being associated with a bite or scratch from an animal that (laboratory) tested positive for rabies.

### Specimen collection, detection and sequencing

**Figure 2:**
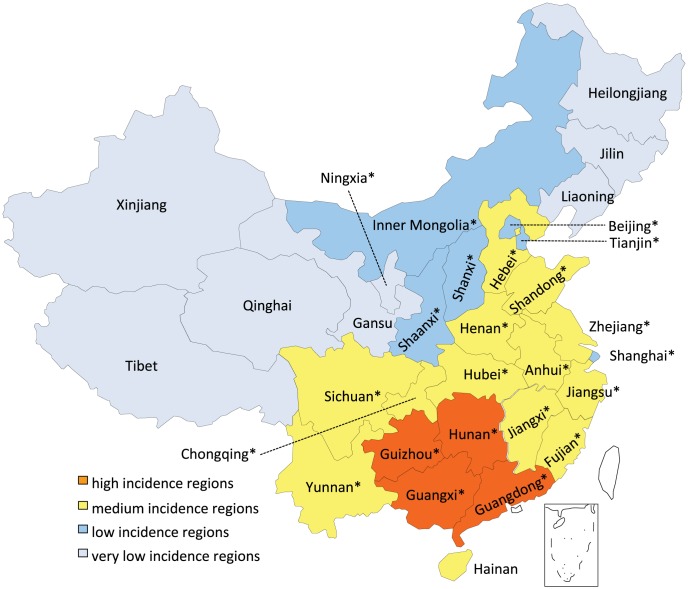
Geographical distribution of rabies cases in China. Based on number of recorded human cases, provinces and regions can be classified into high, medium, low or very low incidence regions. The map shows the provinces are grouped according to incidence and reflects the spread of the epidemic from its origin in south China to the bordering medium incidence regions and out to the low and very low incidence regions. Provinces and municipalities with sequences used in this study are marked with a ‘*’.

Specimens were collected as part of the trial Chinese national surveillance program using the same sampling method as described previously [17]. From 2004 to 2010, 7919 specimens were taken from dog brain, human brain or saliva, and ferret badger (*Melogale moschata*) brain collected in 13 provinces (Hunan, Guangxi, Guizhou, Jiangsu, Zhejiang, Shandong, Shanghai, Anhui, Shaanxi, Jiangxi, Sichuan, Guangdong and Yunnan, Figure 2), and tested for presence of the rabies virus using direct immunofluorescence assay (DFA) [9]. Ferret badgers samples were collected from euthanized animals that had exhibited rabies symptoms and which were found in farming villages. 112 out of all these samples were confirmed positive and the complete G gene coding region (1575 nt) of all the specimens were sequenced by using methods described previously [23]. All sequences were submitted to GenBank and assigned accession numbers as listed in Table S1.

### Dataset for Chinese rabies G sequences

Additionally, all complete G sequences of rabies street strains (as of Jan 6, 2011) were downloaded from GenBank. Only isolates with full background information (isolation time/host/location) were considered and combined with the newly sequenced samples to form a final set of 320 sequences (background information of all sequences are provided in Table S1).

### Dataset for world rabies G sequences

To place the Chinese sequences in perspective with worldwide rabies strains, a second dataset was created that was representative in terms of host, strain and location. A total of 74 sequences were selected, 37 were selected from the China sequences (Table S1) and an additional 37 sequences were selected from the major worldwide lineages (Table S2), representative of countries recording rabies cases in domestic animals, livestock and wildlife.

### Phylogenetic analysis

A maximum clade credibility (MCC) rooted tree was generated and nucleotide substitution rates (per site, per year) were estimated using the Bayesian Markov Chain Monte Carlo (MCMC) methods implemented in the BEAST package (v1.6.2) [24]–[27]. The GTR+I+G model was determined to be the best nucleotide substitution model using ModelTest [28]. The constant population size model was selected based on Bayes Factor and both strict and relaxed (uncorrelated log_normal_) molecular clocks [29] were investigated, and the latter was determined to have the strongest support when results were analyzed in the TRACER program (v1.5), also consistent with previous RABV analyses results [30]. For the world rabies dataset, an NJ tree was constructed with 1000 bootstraps to verify the reliability of the predicted tree.

### Comparison of rabies variant lineages present in China

**Figure 3:**
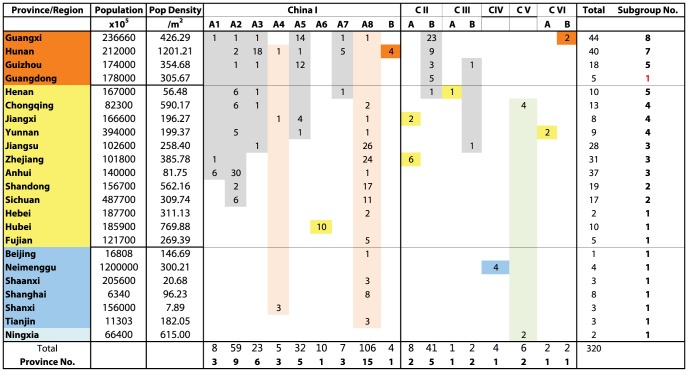
Number of isolates according to province and clade. Provinces are grouped and colour coded according to the high (H), medium (M), low(L) and very low (V) incidence regions defined in Figure 2. The first two columns after the province name show the population and population density for each province. The subsequent columns correspond to the clades and subclades defined in the tree in Figure 4 and show the number of isolates collected for each province and clade/subclade combination. Rightmost column shows the total number of isolates collected for each province. Bottom two rows show the total number of isolates and total number of provinces in each clade/subclade.

The phylogenetic analysis predicted six major clades (China I - China VI) with high statistical support and which appeared to show differences in geographical composition. To investigate the significance of these differences, clades or merged sets of clades were compared to generate contingency tables and a Pearson's Chi-squared test with the Yates' continuity correction was performed. *H_0_*, the null hypothesis, was defined as: *the proportion of cases is independent of column in the contingency table*. *H_A_*, the alternative hypothesis, was defined as: *the proportion of cases is different between columns in the contingency table*. Full details are provided in Table S3. As the China I and China II lineages appeared to be the dominant variant strains, the differences in the geographical composition of these two clades were further investigated. The change in geographical coverage over time was investigated by (i) determining the number of provinces with sequenced isolates assigned to these two clades and (ii) determining the land area encompassed by the provinces with sequenced isolates assigned to these two clades. The land area of each province is given in Figure 3.

### Wildlife dataset

To investigate the contribution from wildlife to rabies in China, wildlife and domestic sequences from the dataset above were combined with the dataset generated from our earlier study based on the N gene to generate a dataset of 368 samples [31]. A complete list of the wildlife isolates are given in Table S4. We then used Fisher's Exact test to identify significant differences between clade pairs. Full details are given in Table S5.

### Analysis of sampling bias

To investigate the possibility of sampling bias, we also investigated the connectivity between human cases (Table S6) and sequenced isolates (Figure 3) by calculating the Pearson's correlation coefficient, according to geographical location (province) and summed over isolation dates (Table S7). The correlation was tested using the Pearson test. We further investigated the connectivity between human cases/isolate numbers and provincial population density. Population density data is taken from national census data and is provided in Figure 3. No provinces reported a significant change in population over the surveillance period.

## Results

### Characteristics and distribution of the current rabies epidemic in China

1996 marked the beginning of a third rabies epidemic in China. From this point the number of human cases increased every year, reaching a peak in 2007. Subsequently, there has been a gradual decrease in the annual cases. Over the course of the epidemic the geographic distribution has spread to encompass almost the entire country and certain characteristics have begun to emerge. Figure 3 (full data is supplied in Table S6) shows the total number of cases by region from 1996 to 2010. Based on this data, we divided mainland China into 4 regions according to the number of human cases. Guangxi, Hunan, Guizhou and Guangdong, located in the South of China, with 4240-2725 cases, were classified as high incidence regions. The medium incidence regions, with 1404-254 cases, were composed of 13 provinces adjacent, or close to, the high incidence regions. The low incidence regions comprised Shanxi, Shaanxi, Inner Mongolia and the three municipalities of Beijing, Tianjin and Shanghai, (96-23 cases) which are located in the north and west of China. The very low incidence regions consisted of Jilin, Liaoning, Heilongjiang, Xinjiang, Gansu, Tibet, Ningxia and Qinghai, all of which are located in the northeast and west of China. These regions are the most distant from the high incidence regions and have experienced almost no human cases in the last 15 years (0–10 cases). Thus, the majority of cases are located in the south of China and there is a gradual decrease in the number of cases towards the north and west of the country.

### Phylogenetic analysis indicates the majority of Chinese rabies cases are associated with two lineages

We next investigated the diversity and relationship amongst Chinese samples by constructing a phylogenetic tree based on our assembled set of G sequences of China street strains. The 320 strains, spanning 1969–2010, were isolated from dogs, humans, deer, mice, cattle, pigs, raccoon dogs and ferret badgers (*Melogale moschata*), and originated from 23 provinces that represent all of the high, medium and low incidence regions shown in Figure 2, with the exception of Hainan province. In addition, samples from Ningxia province, a very low incidence region, were also included.

**Figure 4:**
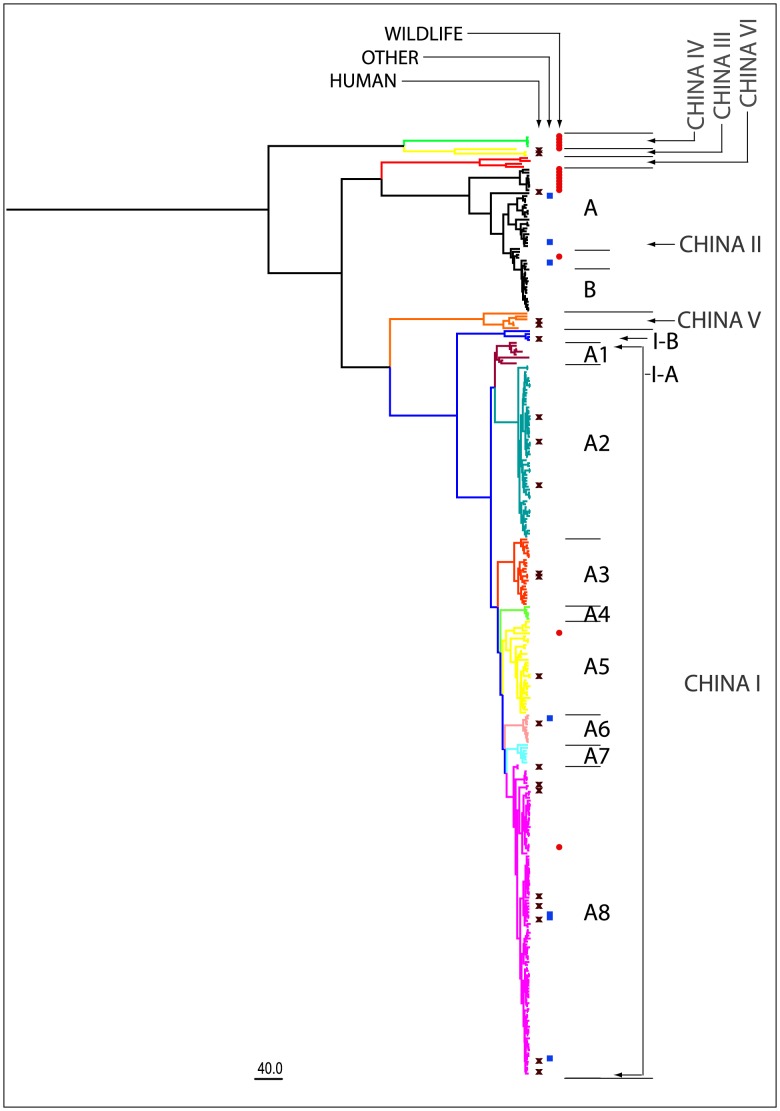
Phylogenetic analysis of China G sequence datasets. The MCC tree of 320 complete G sequences of rabies street strains in China. Consistent with earlier studies, isolates are classified into six major clades China I to VI, with China I and II containing the majority of samples. However, the tree is distinct from previous results in that there are relatively fewer isolates placed in clade II, indicating that China I is now the dominant clade. This clade can be further subdivided into eight major branches with high support. The figure also shows the distribution of hosts throughout the clades. The majority of isolates are from dogs, but human isolates are marked with an X (left column) domesticated animals such as cattle are marked with a blue square (middle column) and wildlife are marked with a circle (right column).

**Figure 5:**
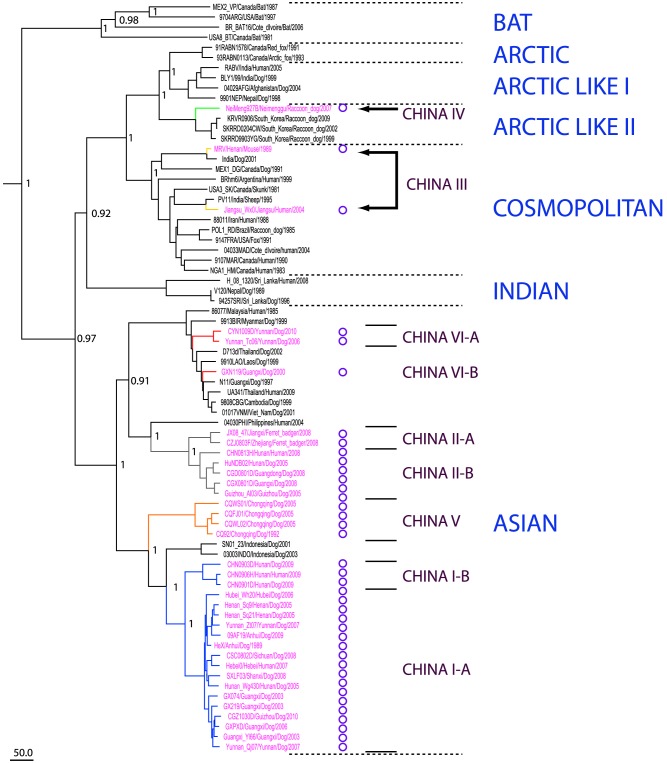
Phylogenetic analysis of World sequence dataset. World tree of representative dataset of 74 world sequences including 37 sequences from the China dataset (these are marked with a circle after the sequence name). The tree shows that China I, II, V & VI are sublineages of the Asian clade, China III corresponds to Cosmopolitan and China IV corresponds to the Arctic-like clade.

The Maximum Clade Credibility (MCC) tree estimated by the BEAST software package is shown in Figure 4. The tree shows China strains form six major lineages or clades, China I to China VI, with high support. Figure 5 shows the relationship of these clades with respect to global lineages. China I, China II, China V and China VI are sub-lineages of the Asian clade, China III corresponds to Cosmopolitan and China IV corresponds to Arctic-like. The limited number of sequences for China III to VI makes it difficult to establish the exact relationship amongst the lineages, but it appears that China I represents the youngest strain in the China tree. However, this lineage is distributed throughout 19 provinces and accounts for more than 80% of all strains (254/320). China II, the second most prevalent clade, includes 49 strains from 7 provinces and accounts for 15% of the samples. The remaining four clades (China III, IV, V, and VI) only account for 5% of the strains, are circulating in limited regions and have little association with the current epidemic (Table S3).

China I can be further divided into subclades I-A and I-B with high support (Figure 4 and Table S1). The I-A subclade contains almost all of the China I strains, and can be further divided into eight branches, also with strong support and are of primary interest. These branches are named A1 to A8 according to their branching order in the tree, with A1 corresponding to the oldest branch and A8 the youngest branch. A8 contains almost half of the China I isolates distributed across 15 provinces, and represents the most important branch from a epidemiological standpoint. The China II clade can also be divided into two subclades II-A and II-B. The 8 ferret badger isolates from Zhejiang and Jiangxi are placed in subclade II-A, and the other 41 strains are placed in subclade II-B and, with the exception of one Henan strain, they all originate from high incidence regions.

### Geographical structure of the estimated tree reflects human case data

To investigate the geographical composition of the sample set according to province or region, the 320 strains were divided according to province/incidence region and the clades, subclades or branches defined in the previous section (Figure 3).

The figure summarizes the composition of each clade, subclade or branch (column) according to province or municipality (row). The dominant role of China I is apparent from the number of samples that are placed in this clade. However, additional patterns are also apparent when considering the relationship amongst the various incidence regions and the defined clades. First of all, the number of clades, subclades or branches associated with each province appears to be greatest for the high incidence regions. The only exception is Guangdong province but this is because only a limited number strains have been made publicly available from this region. Secondly, geographical clustering is evident in the composition of the six China clades. Almost all of the strains from high incidence regions are placed in the China I and China II clades. The II-B strains originate from high incidence regions, with the exception of a Henan strain isolated in 1993, suggesting that this subclade circulated in the early phase of the third epidemic, but failed to spread further as the epidemic became more established.

The China I branches show a more complex pattern of geographical composition. Branches I-A3, I-A5 and I-A7 are primarily composed of isolates from high incidence regions, but also contain several isolates from medium incidence regions. The remaining branches show increased mixing of isolates from high, medium and low incidence regions. I-A8, the youngest branch, contains the largest number of sequences and these were isolated from high, medium and low incidence regions, but with the most isolates collected from the medium incidence regions and the fewest isolates collected from high incidence regions.

To investigate whether the differences in the geographical composition of the branches were significant, we compared branch I-A8 and to the combined set of strains from I-A1 to A7 and performed a statistical comparison as described in the Materials and Methods. Our results indicate that an extremely significant difference exists in the proportions of isolates from high, medium and low incidence regions for I-A8 and the remaining branches (P = 9.126×10^−14^). This difference extends to the differences in the proportions of low to medium, and medium to high for these branches (Full details of the analysis are given in Table S3). Thus, the composition of the branches within the China I-A subclade reflects the observed spread of human cases over time from high to medium and low incidence regions. The older clades are primarily composed of sequences from high incidence regions. The primary exception to this pattern is the isolation of samples from the low case region of Neimenggu (Inner Mongolia) that are placed in the China IV/Arctic-like clade. This suggests that this lineage plays a minor role in the epidemic, further supported by our recently published results investigating the relationship amongst China rabies and lineages in neighbouring countries [31].

### The number of human cases continued to increase in medium and low incidence regions after the number of national cases decreased

**Figure 6:**
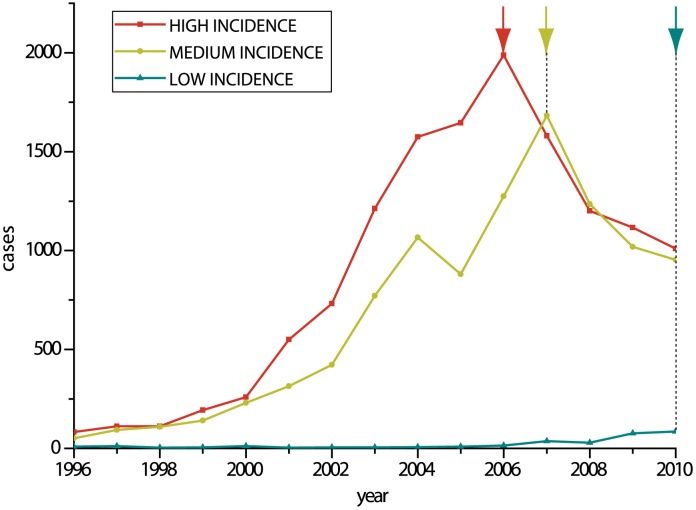
Number of rabies cases in high, medium and low incidence regions, 1996–2010. The graph shows the same data as figure 1, but according to the high, medium and low incidence regions defined in figure 2. The graph shows that while the number of cases in the high incidence regions peaked in 2006, consistent with national data, the number of cases in the medium incidence regions continued to rise for another year (marked by arrows at the top of the graph). The number of cases in low incidence regions continues to rise.

The classification of human case numbers into high, medium, low and very low incidence regions was based on data collected nationally between 1996 to 2010 (Figure 1 - third wave). Figure 6 shows the same data for the third wave but broken down into the number of cases for the high, medium and low incidence regions. Coincident with cases at the national level, the numbers of cases for the high incidence regions peaked in 2006 and then began to decrease gradually. For the medium incidence regions, cases peaked a year later in 2007 and then began to decrease in accordance with the high incidence regions. However, for the low incidence regions, there were almost no cases until 2007 but after this point the number of cases began to increase at a similar rate to that seen at the beginning of the third wave in the high incidence regions. The maximum number of cases occurred in 2010 and it seems numbers will continue to increase. Thus, over the course of the epidemic the burden appears to have shifted from the high to medium and low incidence regions and is now encroaching into regions which have until recently recorded few events.

### China I has emerged to become the dominant lineage in the current rabies epidemic

**Figure 7:**
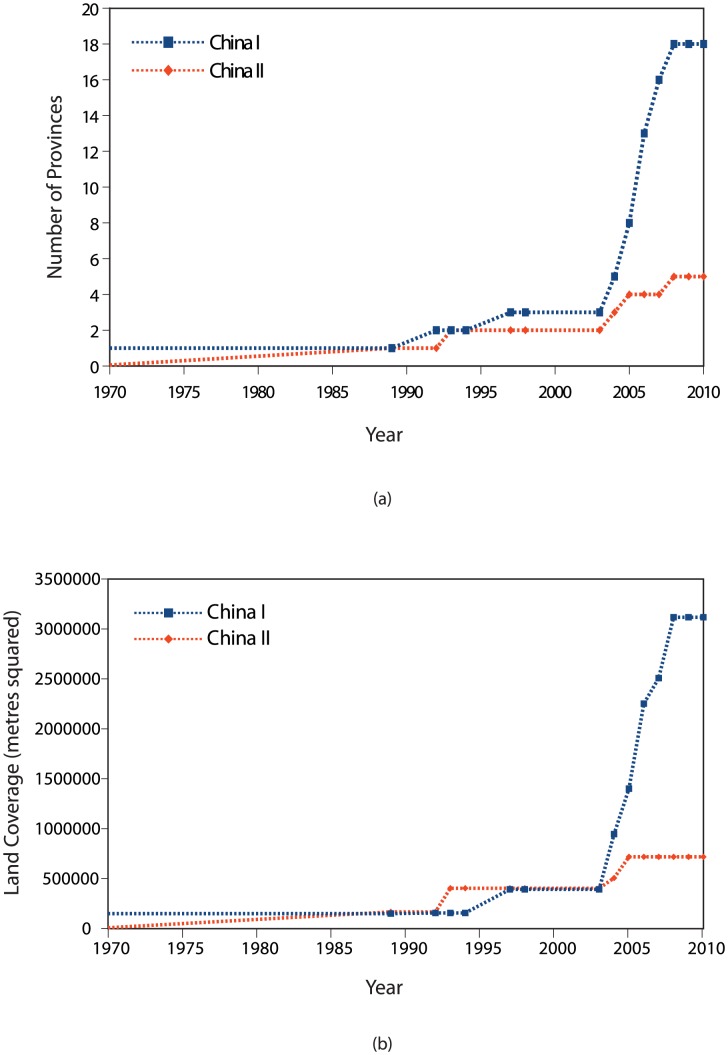
Change in geographical dispersion of China I and China II lineages over time. Graphs show the change in geographical coverage over time for the two major lineages China I and China II lineages over the course of the current epidemic. Coverage was measured in terms of (a) number of provinces reporting each lineage and (b) total land area in square metres based on the summed area of the provinces reporting each lineage. In the early stages of the epidemic, the two clades showed similar dispersion patterns, but after 2005 China I rapidly expanded whereas China II isolates were only collected in existing regions.

To further examine the contributions of the China I and II lineages to the current epidemic, we next investigated the change in their geographical distribution over the course of the epidemic. Graphs of the number of provinces reporting isolates from these two lineages and the total land area for reporting provinces are shown in Figure 7a and 7b respectively. These figures show that in the early stages of the current epidemic, the two lineages spread into new regions at similar rates but, after 2004, China I underwent a rapid geographic expansion whereas China II remained within its existing region of influence, further highlighting the dominance of the former lineage.

### Wildlife isolates are almost exclusively isolated with older lineages

The two major lineages also show differences in their host composition (Figure 4). Despite the geographic expansion of China I and the association of the majority of new isolates with this lineage, the samples were isolated almost exclusively from dogs, with almost all the remaining samples collected from humans and domesticated animals. Only two samples were isolated from wildlife (deer and ferret badger). Conversely, for the China II, III and IV lineages, a larger proportion of isolates were collected from wildlife (Table 1). No wildlife isolates were collected for lineages China V and VI, but the total number of isolates were small for both these clades. Pairwise comparison of the China I, II, III and IV lineages using the Fisher's Exact test revealed that China I had a distinct host composition from China II (P = 2.74×10^−9^), whereas the composition of China II and China III (Cosmopolitan) were indistinguishable (P = 1). However, a significance difference was predicted between China III and China IV (Arctic-like clade) (P = 0.0048). Full details of the statistical tests are given in Table S5.

**Table 1:** Number of wildlife versus dog isolates for lineages China I to China IV.

Clade	Wildlife strains	Dog strains	Total strains
China I	2	272	299
China II	15	60	83
China III (Cosmopolitan)	3	11	16
China IV (Arctic-like)	5	0	5

### Sample isolation reflects the epidemic

**Figure 8:**
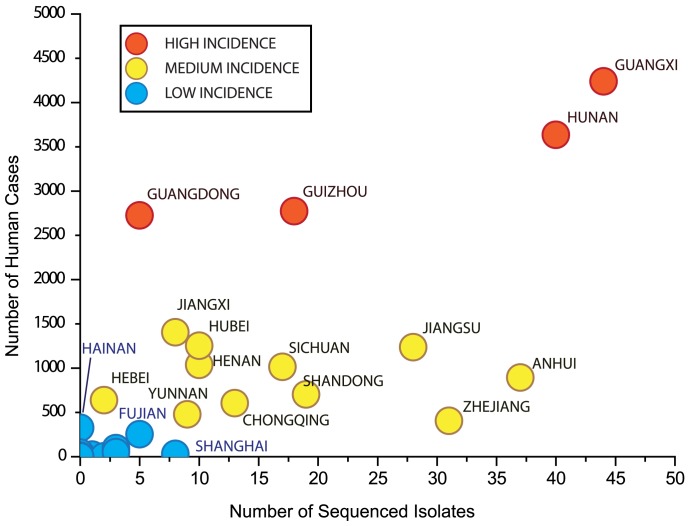
Investigation of sampling accuracy. Graph shows total number of isolates collected for each province versus the corresponding total number of human rabies over the course of the current epidemic.

One of the goals of the current national rabies surveillance program is the collection of isolates from regions where rabies cases have been reported in order to obtain a characteristic dataset. To investigate whether the collection program is a reasonable representation of the current epidemic, we compared the total number of isolates collected from each province to the corresponding total number of human cases recorded for that province (Table S7). The calculated Pearson's correlation coefficient was *r* = 0.7189 (p-value: 3.557×10^−06^) indicating the presence of a strong correlation and that, overall, the collected isolates were representative of the current rabies epidemic in China (Figure 8). We also compared the number of collected isolates to the population and the population density of each province respectively. We found a weaker but significant correlation for provincial population versus sampling/number of cases (*r* = 0.4801772, p = 0.006/*r* = 0.4596770, p = 0.009). This reflects that many of the medium and high case regions represent some of the largest provincial populations. It is more informative that there was no measurable correlation between population density and sampling/number of cases (*r* = −0.08634058, p = 0.6442/*r* = 0.04543189, p = 0.8083)- i.e, highly populated regions, including major cities such as Beijing, Shanghai or Chongqing with better resources, are not being preferentially sampled.

## Discussion

In the face of the most recent rabies epidemic, the Chinese government implemented a trial national surveillance program in an attempt to gain a better understanding of the mechanisms driving the reemergence of the disease. Previous studies have revealed evidence of epidemic waves in other countries that are proposed to be synchronous with and in response to vaccination programs, as well as revealing the role of humans in the dispersion of the virus [32], [33], but this is the most comprehensive study to date in terms of the size of the geographic region and a time period that spanned the presence and absence of vaccination and other control programs.

Traditionally, rabies epidemics have been tracked in terms of surveillance data that presents a statistic such as number of human cases. With advances in analytical techniques and the extensive virus sequence data obtained from the trial surveillance program (as well as other sequences submitted to GenBank) it is now possible to investigate the genetic diversity of the variant strains to learn about the origins, expansion and evolutionary dynamics of the current epidemic. In this work, we analyzed a comprehensive sequence set covering almost all epidemic regions in China. While the identification of 6 major RABV lineages is consistent with other results from recent studies, it is the change in the composition of these clades that is informative. In 2007, only 3 RABV lineages were found in the south of China [9], but by 2008, rabies virus samples collected from 15 provinces were associated with 4 distinct lineages, all of which were present in the south of the country [17]. Current reports now indicate there are now 6 lineages of RABV [20], [23].

The emergence of the China I lineage and the gradual displacement of China II is also apparent when comparing our dataset with those used in other recent studies [9], [17], [20]. Whereas there were comparable numbers of China I and China II strains in the earlier study, almost all the strains collected in this study in 2009 and 2010 from 9 provinces belonged to the China I lineage (Table S1). Furthermore, the geographical dispersion patterns determined in our analysis are consistent with the patterns observed in human case data. Both datasets show the south provinces are the source of the current outbreak and the number of human cases decreases according to geographical distance from this region.

Our phylodynamic analysis reveals additional patterns and characteristics of the current epidemic. Figure 7 shows how rapidly China I displaced China II and gained dominance. Furthermore, comparison with Figure 6 suggests that the recent cases in previously rabies free provinces are associated with this lineage; the number of human cases in high and medium case regions began to decrease in 2006 and 2007 respectively, but this coincided with the rapid expansion of China I. Also, as China I was becoming established, the remaining lineages China III to VI were playing a progressively less significant role in the epidemic. In contrast to the second wave and early stages of the third wave, only a few strains from these clades have been isolated in recent years and have been located within a narrow geographic range, suggesting that some of them may be disappearing.

The significant differences in the host range of the lineages is also informative. China I isolates are almost exclusively from dogs, domestic animals and human cases, whereas China II, III and IV contains a far higher proportion of wildlife isolates; it is only recently that China I strains have been isolated in wildlife. This data appears to highlight the fundamental role of dogs in the spread of the disease as well as the dissemination of a new lineage and it appears the spillover into wildlife only occurs once the lineage is well established.

The phylogenetic analysis, together with the observation that lineages China II, China III and IV are established in wildlife, suggests that they were dominant in earlier epidemics. However, the greater number of China II isolates compared to these other lineages and the observed displacement of this lineage in recent years suggests that China II was dominant in the second epidemic and that another lineage (possibly China III, V or VI) was dominant in the first epidemic. It seems unlikely that China IV has played a significant role in the major rabies epidemics in China (in terms of dog and human cases) as isolates are few and restricted to the north west of the country, and most of the isolates were obtained from wildlife. The rapid emergence of China I indicates that, although it was present in previous epidemics, this lineage is responsible for the current epidemic. Figure S1 summarizes the temporal, geographical and phylogenetic classification of the various strains. In particular, the figure shows the minimal contribution from lineages China III, IV, V & VI in the current epidemic, and how successive sub-lineages of China I gradually spread across the country while China II remained constrained to the southwestern provinces.

These dispersion patterns cannot be attributed to any identified sampling bias as our statistical analysis indicate the isolates selected for sequencing and used in this analysis are highly representative of the national situation according to human cases. Also, we identified no correlation between number of isolates and provincial population density. This suggests that the current surveillance program, based on standard reporting protocols at the local level and coordination at the national level, is effective at capturing the current rabies situation in China. The notable exception is Guangdong province, which is a high case region, but few sequences have been made publicly available. It is hoped that this situation will change in the near future so that the rabies situation in this province can be fully evaluated.

One important distinction between the current and earlier epidemics is the methods that were implemented to try and bring the outbreak under control. In the first two epidemics, in the absence of widespread vaccination programs, dog culling and restrictions on dog ownership (Notice for strengthening work on rabies prevention and control. 1984. http://law.lawtime.cn/d564741569835.html) were the primary methods for containing the virus [34]. As discussed above, recordkeeping was not as precise in the earlier epidemics, but the general features of the graph in figure 1 nevertheless highlight the limitations of culling as an effective control measure. Although the widespread culling was implemented in the middle of the second epidemic, it was more than ten years before the number of cases was brought under control. Furthermore, the numbers actually increased after the introduction of a nationwide culling program. While the reasons for this are not entirely clear, it may well have been a consequence of the implementation of a national culling program. Previously, dog culling efforts were in place, but they were directed by provincial or city government and there was a delay before nationally coordinated program could become functional. Furthermore, our analysis of the geographical and temporal dispersion of RABV strains indicates that, rather than producing widespread eradication of hosts in a short time, it resulted in a number of isolated strains in specific regions. When the culling program was finally halted, there were several strains circulating which were able to compete for dominance. Of these, the China I lineage was able to emerge, while the other lineages remained within their limited geographic range. This also helps us to understand the relatively low estimates for the TMRCA of rabies in China. Despite records of the disease extending back more than 2500 years, estimates based on coalescence date the origin to no more than a few hundred years [20], [21]. The common explanation is the original lineage has died out and been replaced by newer ones [30], our results showing the emergence of China I in the most recent epidemic is the first evidence that this does indeed occur.

The expansion of the epidemic into low incidence regions is a cause for concern. Prior to 2007, there were less than 10 human rabies cases reported in these regions in the last 10 years (Table S3). Since then, the number of human cases in these regions has increased as the number of cases have begun to decrease at a national level [23]. New strains isolated in 2011 in Shanxi, Inner Mongolia and Ningxia are genetically close I-A8 strains [23], indicating that this lineage is already becoming established throughout the low incidence regions and is beginning to expand into very low incidence regions. This is also supported by data that has been collected after the reported study period. In 2011, the reported cases in Inner Mongolia, Shanxi, Shaanxi and Shanghai, corresponding to low incidence regions (Figure 3), doubled over the previous year. Moreover, Ningxia, Xinjiang, Liaoning and Heilongjiang provinces, which have had no cases reported in recent years (Table S3), began to report their first cases in 2011 or 2012.

The rapid emergence of a third epidemic wave so rapidly after the second epidemic had been controlled highlights the challenge of completely eradicating rabies. The geography of high incidence regions such as Guangxi, Hunan and Guizhou makes it possible for disease reservoirs to exist in remote or inaccessible regions and the custom of eating dog meat ensures the continual presence of localized concentrations of dog populations, which further benefits the survival of RABV [9]. On a more positive note, our analysis shows that the recent introduction of alternative control measures are proving effective in combating the spread of the disease and reducing the number of human cases. In particular, trial dog vaccination programs implemented in some high case regions in the southwest provinces have highlighted the effectiveness of vaccination for rabies control. These successes are reflected in the recent announcement of a new draft plan for rabies control by the Ministry of Agriculture and Health. These new regulations place emphasis on rabies control at the source, emphasizing vaccination of domestic animals, especially in rural areas. In the next phase of the program, vaccination will be extended to additional regions to incorporate more of the dog population. Furthermore, the integration of postexposure prophylaxis costs into welfare programs combined with rabies education as well as introduction of animal registration policies yielded a reduction in the number of cases at the national level within two years. This highlights the importance of continuing these programs as well as establishing similar programs in low incidence regions before the disease can become further established, leading to a reemergence of human cases.

## Supporting Information

Figure S1 **Geographical and temporal composition of the six China clades and branches defined according to the trees in** **Figure 4** **and** **Figure 6** **.** Each subclade or branch is summarized as set of circles in one column, with each circle corresponding to the data collected for one province for that clade. The inner and outer radius of each circle correspond to the earliest and latest collection times for that province. For example, the vertical arrow marks the circle corresponding to data collected for Anhui province for China I branch A1. Top Right. The solid circle indicates the Anhui samples are composed of the earliest samples in the entire dataset (1969), and the smaller outer radius (compared to almost all the other circles) indicates the latest data was collected before most of the other data in the dataset (1989). Bottom Right. Conversely, the horizontal arrow marks the data collected for China I-A6 for Shandong province. This is a larger diameter circle, but there is less solid color, so the data has been collected at a later time, but within a relatively limited time period (2005–2008). The circles in the older clades (II, III, IV, IV, VI) generally have smaller circle suggesting these clades were rarely sampled in the current epidemic. Also, in these older clades, the circles are very close together on the y-axis, indicating limited geographical dispersal. Conversely, the circles in the newer China I generally have larger circles with a narrow line. This means they are commonly sampled in the current epidemic, but were not sampled in the previous epidemics. Additionally, the circles are more spread out on the y-axis indicating greater geographical dispersal. For branches A2 to A8, there is gradual increase in the geographical dispersal, consistent with the observed spread of the epidemic.
(EPS)

Table S1 **Background information for the 320 G nucleotide sequences used in this study. Sequences are grouped according to their assigned clade in the tree shown in** **Figure 3** **.** New sequences collected in this study are marked with a ‘+’ in the column second from right. Sequences used in the World tree (Figure 4) are marked with a ‘*’ in the rightmost column.
(DOC)

Table S2 **Background information of extra sequences used in the World tree (** **Figure 4** **).**
(DOC)

Table S3 **The Pearson's Chi-squared test results for the differences in geographical composition.**
(DOC)

Table S4 **Background information of wildlife sequences collected from N and G sequences from this study and previously submitted to GenBank.**
(DOC)

Table S5 **Comparison of dog and wildlife composition of Chinese rabies lineages.**
(DOC)

Table S6 **The numbers of human cases in each province or municipality of China from 1996 to 2010.**
(DOC)

Table S7 **Number of collected isolates versus number of human rabies cases by province.**
(DOC)
