# Supplementary material for: Molecular Phylodynamic Analysis Indicates Lineage Displacement Occurred in Chinese Rabies Epidemics between 1949 to 2010
Source: PLoS Negl Trop Dis. 2013 Jul 11;7(7):e2294. doi: 10.1371/journal.pntd.0002294 (PMC3708843; doi:10.1371/journal.pntd.0002294)
Supplement: Table S1 — Background information for the 320 G nucleotide sequences used in this study. Sequences are grouped according to their assigned clade in the tree shown in Figure 3 . New sequences collected in this study are marked with a ‘+’ in the column second from right. Sequences used in the World tree (Figure 4) are marked with a ‘*’ in the rightmost column. (DOC) [file pntd.0002294.s002.doc]

**Supplementary Table S1:** background information for the 320 G nucleotide sequences used in this study. New sequences collected in this study are marked with a '+' in the column second from right. Sequences used in World tree are marked with a '*' in the rightmost column

|  | **Group** | **GenBank No.** | **Strain** | **Region** | **Host** | **Year** |  |  |
| --- | --- | --- | --- | --- | --- | --- | --- | --- |
| 1 | China I-A1 | DQ849073 | FEIDONG | Anhui | Dog | 1989 |  |  |
| 2 | China I-A1 | FJ418879 | FS | Guangxi | Dog | 1998 |  |  |
| 3 | China I-A1 | FJ418881 | H69 | Anhui | Dog | 1969 |  |  |
| 4 | China I-A1 | FJ418882 | H | Anhui | Dog | 1989 |  |  |
| 5 | China I-A1 | FJ602450 | HeFei | Anhui | Dog | 1989 |  |  |
| 6 | China I-A1 | FJ602459 | HeX | Anhui | Dog | 1989 |  | * |
| 7 | China I-A1 | GQ857471 | WJ | Zhejiang | Dog | 2008 |  |  |
| 8 | China I-A1 | GQ857472 | H89 | Anhui | Dog | 1989 |  |  |
| 9 | China I-A2 | JN936698 | CGZ0515D | Guizhou | Dog | 2005 | + |  |
| 10 | China I-A2 | JN936708 | CHN0602D | Hunan | Dog | 2006 | + |  |
| 11 | China I-A2 | JN936710 | CHN0614D | Hunan | Dog | 2006 | + |  |
| 12 | China I-A2 | JN936752 | CSC0808D | Sichuan | Dog | 2008 | + |  |
| 13 | China I-A2 | JN936753 | CSC0909D | Sichuan | Dog | 2009 | + |  |
| 14 | China I-A2 | JN936754 | CSC0910D | Sichuan | Dog | 2009 | + |  |
| 15 | China I-A2 | JN936755 | CSC0911D | Sichuan | Dog | 2009 | + |  |
| 16 | China I-A2 | JN936757 | CSC1013D | Sichuan | Dog | 2010 | + |  |
| 17 | China I-A2 | JN936763 | CSC1019H | Sichuan | Human | 2010 | + |  |
| 18 | China I-A2 | JN936771 | CSD0805D | Shandong | Dog | 2008 | + |  |
| 19 | China I-A2 | JN936776 | CSD0934D | Shandong | Dog | 2009 | + |  |
| 20 | China I-A2 | JQ040578 | CYN0919D | Yunnan | Dog | 2009 | + |  |
| 21 | China I-A2 | JF819602 | CYN1004D | Yunnan | Dog | 2010 | + |  |
| 22 | China I-A2 | JF819600 | CYN1005D | Yunnan | Dog | 2010 | + |  |
| 23 | China I-A2 | JF819598 | CYN1007H | Yunnan | Human | 2010 | + |  |
| 24 | China I-A2 | DQ849044 | FY1 | Anhui | Dog | 2004 |  |  |
| 25 | China I-A2 | DQ849045 | FY2 | Anhui | Dog | 2004 |  |  |
| 26 | China I-A2 | DQ849046 | FY3 | Anhui | Dog | 2004 |  |  |
| 27 | China I-A2 | DQ849047 | FY4 | Anhui | Dog | 2004 |  |  |
| 28 | China I-A2 | DQ849048 | FY5 | Anhui | Dog | 2004 |  |  |
| 29 | China I-A2 | DQ849049 | FY6 | Anhui | Dog | 2004 |  |  |
| 30 | China I-A2 | DQ849050 | FY7 | Anhui | Dog | 2004 |  |  |
| 31 | China I-A2 | DQ849051 | FY8 | Anhui | Dog | 2004 |  |  |
| 32 | China I-A2 | DQ849052 | FY9 | Anhui | Dog | 2004 |  |  |
| 33 | China I-A2 | DQ849053 | FY10 | Anhui | Dog | 2004 |  |  |
| 34 | China I-A2 | DQ849054 | FY12 | Anhui | Dog | 2005 |  |  |
| 35 | China I-A2 | DQ849055 | FY13 | Anhui | Dog | 2005 |  |  |
| 36 | China I-A2 | DQ849056 | FY14 | Anhui | Dog | 2004 |  |  |
| 37 | China I-A2 | DQ849057 | FY15 | Anhui | Dog | 2005 |  |  |
| 38 | China I-A2 | DQ849058 | FY16 | Anhui | Dog | 2005 |  |  |
| 39 | China I-A2 | EU267742 | Guangxi Cx14 | Guangxi | Dog | 2003 |  |  |
| 40 | China I-A2 | EU267753 | Henan Hb10 | Henan | Dog | 2005 |  |  |
| 41 | China I-A2 | EU267755 | Henan Sq9 | Henan | Dog | 2005 |  | * |
| 42 | China I-A2 | EU267756 | Henan Sq10 | Henan | Dog | 2005 |  |  |
| 43 | China I-A2 | EU267757 | Henan Sq21 | Henan | Dog | 2005 |  | **X** |
| 44 | China I-A2 | EU267759 | Henan Sq59 | Henan | Dog | 2005 |  |  |
| 45 | China I-A2 | EU275241 | Yunnan_Zt07 | Yunnan | Dog | 2007 |  | ***** |
| 46 | China I-A2 | FJ602453 | LuoH | Henan | Human | 2007 |  |  |
| 47 | China I-A2 | GQ857450 | 09AF19 | Anhui | Dog | 2009 |  | ***** |
| 48 | China I-A2 | GQ857451 | 09AF20 | Anhui | Dog | 2009 |  |  |
| 49 | China I-A2 | GQ857452 | 09AF21 | Anhui | Dog | 2009 |  |  |
| 50 | China I-A2 | GQ857453 | 09AF22 | Anhui | Dog | 2009 |  |  |
| 51 | China I-A2 | GQ857454 | 09AF23 | Anhui | Dog | 2009 |  |  |
| 52 | China I-A2 | GQ857455 | 09AF24 | Anhui | Dog | 2009 |  |  |
| 53 | China I-A2 | GQ857456 | 09AF25 | Anhui | Dog | 2009 |  |  |
| 54 | China I-A2 | GQ857457 | 09AF26 | Anhui | Dog | 2009 |  |  |
| 55 | China I-A2 | GQ857458 | 09AF27 | Anhui | Dog | 2009 |  |  |
| 56 | China I-A2 | GQ857459 | 09AF28 | Anhui | Dog | 2009 |  |  |
| 57 | China I-A2 | GQ857460 | 09AF29 | Anhui | Dog | 2009 |  |  |
| 58 | China I-A2 | GQ857461 | 09AF30 | Anhui | Dog | 2009 |  |  |
| 59 | China I-A2 | GQ857462 | 09AF31 | Anhui | Dog | 2009 |  |  |
| 60 | China I-A2 | GQ857463 | 09AF32 | Anhui | Dog | 2009 |  |  |
| 61 | China I-A2 | GQ857464 | 09AF33 | Anhui | Dog | 2009 |  |  |
| 62 | China I-A2 | GU186390 | CQQJ07 | Chongqing | Dog | 2007 |  |  |
| 63 | China I-A2 | GU186391 | CQQJ08 | Chongqing | Dog | 2008 |  |  |
| 64 | China I-A2 | GU186392 | CQQJ09 | Chongqing | Dog | 2008 |  |  |
| 65 | China I-A2 | GU186393 | CQQJ10 | Chongqing | Dog | 2008 |  |  |
| 66 | China I-A2 | GU186394 | CQQJ11 | Chongqing | Dog | 2008 |  |  |
| 67 | China I-A2 | GU186395 | CQQJ12 | Chongqing | Dog | 2008 |  |  |
| 68 | China I-A3 | JN936709 | CHN0610H | Hunan | Human | 2006 | + |  |
| 69 | China I-A3 | JN936712 | CHN0635H | Hunan | Human | 2006 | + |  |
| 70 | China I-A3 | JN936714 | CHN0701D | Hunan | Dog | 2007 | + |  |
| 71 | China I-A3 | JN936716 | CHN0808D | Hunan | Dog | 2008 | + |  |
| 72 | China I-A3 | JN936717 | CHN0809D | Hunan | Dog | 2008 | + |  |
| 73 | China I-A3 | JN936718 | CHN0810D | Hunan | Dog | 2008 | + |  |
| 74 | China I-A3 | DQ849059 | WG432 | Hunan | Dog | 2005 |  |  |
| 75 | China I-A3 | DQ849060 | WG430 | Hunan | Dog | 2005 |  |  |
| 76 | China I-A3 | EU267743 | Guangxi Cx25 | Guangxi | Dog | 2003 |  |  |
| 77 | China I-A3 | EU267748 | Guizhou Al48 | Guizhou | Dog | 2004 |  |  |
| 78 | China I-A3 | EU267758 | Henan Sq35 | Henan | Dog | 2005 |  |  |
| 79 | China I-A3 | EU267762 | Hunan Dk13 | Hunan | Dog | 2004 |  |  |
| 80 | China I-A3 | EU267763 | Hunan Wg12 | Hunan | Dog | 2004 |  |  |
| 81 | China I-A3 | EU267764 | Hunan Wg13 | Hunan | Dog | 2004 |  |  |
| 82 | China I-A3 | EU267765 | Hunan Wg26 | Hunan | Dog | 2004 |  |  |
| 83 | China I-A3 | EU267766 | Hunan Wg27 | Hunan | Dog | 2004 |  |  |
| 84 | China I-A3 | EU267767 | Hunan Wg430 | Hunan | Dog | 2005 |  | ***** |
| 85 | China I-A3 | EU267768 | Hunan Wg432 | Hunan | Dog | 2004 |  |  |
| 86 | China I-A3 | EU267774 | Jiangsu Wx32 | Jiangsu | Dog | 2005 |  |  |
| 87 | China I-A3 | GU186381 | HuNDB06 | Hunan | Dog | 2006 |  |  |
| 88 | China I-A3 | GU186382 | HuNDB07 | Hunan | Dog | 2007 |  |  |
| 89 | China I-A3 | GU186383 | HuNDB16 | Hunan | Dog | 2005 |  |  |
| 90 | China I-A3 | GU186388 | CQFJ02 | Chongqing | Dog | 2007 |  |  |
| 91 | China I-A4 | JN936706 | CHN0505D | Hunan | Dog | 2005 | + |  |
| 92 | China I-A4 | JN936746 | CJX0903D | Jiangxi | Dog | 2009 | + |  |
| 93 | China I-A4 | GU186406 | SXAZ01 | Shanxi | Dog | 2007 |  |  |
| 94 | China I-A4 | GU186407 | SXAZ02 | Shanxi | Dog | 2007 |  |  |
| 95 | China I-A4 | GU186408 | SXLF03 | Shanxi | Dog | 2008 |  | ***** |
| 96 | China I-A5 | JN936694 | CGX0626D | Guangxi | Dog | 2006 | + |  |
| 97 | China I-A5 | JN936696 | CGZ0501D | Guizhou | Dog | 2005 | + |  |
| 98 | China I-A5 | JN936699 | CGZ0622D | Guizhou | Dog | 2006 | + |  |
| 99 | China I-A5 | JN936700 | CGZ0623D | Guizhou | Dog | 2006 | + |  |
| 100 | China I-A5 | JN936701 | CGZ1025D | Guizhou | Dog | 2010 | + |  |
| 101 | China I-A5 | JN936702 | CGZ1026H | Guizhou | Human | 2010 | + |  |
| 102 | China I-A5 | JN936703 | CGZ1029D | Guizhou | Dog | 2010 | + |  |
| 103 | China I-A5 | JN936704 | CGZ1030D | Guizhou | Dog | 2010 | + | ***** |
| 104 | China I-A5 | JN936744 | CJX0901D | Jiangxi | Dog | 2009 | + |  |
| 105 | China I-A5 | JN936747 | CJX0906D | Jiangxi | Dog | 2009 | + |  |
| 106 | China I-A5 | DQ849064 | NC | Jiangxi | Dog | 2004 |  |  |
| 107 | China I-A5 | DQ849070 | YUE1 | Guangxi | Dog | 1997 |  |  |
| 108 | China I-A5 | EU267744 | Guangxi Yl66 | Guangxi | Dog | 2003 |  | ***** |
| 109 | China I-A5 | EU267749 | Guizhou Qx1 | Guizhou | Dog | 2004 |  |  |
| 110 | China I-A5 | EU267750 | Guizhou Qx2 | Guizhou | Dog | 2004 |  |  |
| 111 | China I-A5 | EU267751 | Guizhou Qx5 | Guizhou | Dog | 2004 |  |  |
| 112 | China I-A5 | EU275240 | Yunnan_Qj07 | Yunnan | Dog | 2007 |  | ***** |
| 113 | China I-A5 | FJ418878 | Yue2 | Guangxi | Dog | 1997 |  |  |
| 114 | China I-A5 | FJ418885 | gk5 | Guizhou | Dog | 2006 |  |  |
| 115 | China I-A5 | FJ418887 | gg4 | Guizhou | Dog | 2006 |  |  |
| 116 | China I-A5 | GQ472539 | GX074 | Guangxi | Dog | 2003 |  | ***** |
| 117 | China I-A5 | GQ472542 | GX219 | Guangxi | Dog | 2003 |  | ***** |
| 118 | China I-A5 | GQ472544 | GX304 | Guangxi | Dog | 2004 |  |  |
| 119 | China I-A5 | GQ472545 | GXBM | Guangxi | Dog | 2003 |  |  |
| 120 | China I-A5 | GQ472548 | GXLA11 | Guangxi | Dog | 2007 |  |  |
| 121 | China I-A5 | GQ472551 | GXLCC | Guangxi | Dog | 2007 |  |  |
| 122 | China I-A5 | GQ472553 | GXNN2 | Guangxi | Dog | 2007 |  |  |
| 123 | China I-A5 | GQ472555 | GXPL | Guangxi | Dog | 2007 |  |  |
| 124 | China I-A5 | GQ472556 | GXPXD | Guangxi | Dog | 2006 |  | ***** |
| 125 | China I-A5 | GQ472560 | GXYZD | Guangxi | Dog | 2007 |  |  |
| 126 | China I-A5 | GU186384 | HuNDB28 | Hunan | Dog | 2005 |  |  |
| 127 | China I-A5 | GU233763 | JX09-17(fb) | Jiangxi | Ferret badger | 2009 |  |  |
| 128 | China I-A6 | DQ849061 | WH5 | Hubei | Dog | 2005 |  |  |
| 129 | China I-A6 | DQ849062 | HN06 | Hubei | Dog | 2005 |  |  |
| 130 | China I-A6 | DQ849063 | QC | Hubei | Human | 2006 |  |  |
| 131 | China I-A6 | EU267760 | Hubei Wh20 | Hubei | Dog | 2006 |  | ***** |
| 132 | China I-A6 | EU267761 | Hubei Wh22 | Hubei | Dog | 2006 |  |  |
| 133 | China I-A6 | FJ602449 | HByx | Hubei | Dog | 2008 |  |  |
| 134 | China I-A6 | FJ602451 | HNC | Hubei | Cattle | 2007 |  |  |
| 135 | China I-A6 | FJ602454 | WHqs | Hubei | Dog | 2007 |  |  |
| 136 | China I-A6 | FJ602455 | WHWD | Hubei | Dog | 2007 |  |  |
| 137 | China I-A6 | FJ602456 | WHyf | Hubei | Dog | 2007 |  |  |
| 138 | China I-A7 | JN936691 | CGX0603D | Guangxi | Dog | 2006 | + |  |
| 139 | China I-A7 | JN936705 | CHN0503D | Hunan | Dog | 2005 | + |  |
| 140 | China I-A7 | EU267754 | Henan Sq6 | Henan | Dog | 2005 |  |  |
| 141 | China I-A7 | EU267769 | Hunan Xx33 | Hunan | Dog | 2004 |  |  |
| 142 | China I-A7 | EU267770 | Hunan Xx34 | Hunan | Dog | 2004 |  |  |
| 143 | China I-A7 | EU267771 | Hunan Xx35 | Hunan | Dog | 2004 |  |  |
| 144 | China I-A7 | GU186385 | HuNDB33 | Hunan | Dog | 2006 |  |  |
| 145 | China I-A8 | JN936687 | CAH0501D | Anhui | Dog | 2005 | + |  |
| 146 | China I-A8 | JN936692 | CGX0606D | Guangxi | Dog | 2006 | + |  |
| 147 | China I-A8 | JN936725 | CJS0523D | Jiangsu | Dog | 2005 | + |  |
| 148 | China I-A8 | JN936726 | CJS0538D | Jiangsu | Dog | 2005 | + |  |
| 149 | China I-A8 | JN936727 | CJS0621D | Jiangsu | Dog | 2006 | + |  |
| 150 | China I-A8 | JN936728 | CJS0634D | Jiangsu | Dog | 2006 | + |  |
| 151 | China I-A8 | JN936729 | CJS0635D | Jiangsu | Dog | 2006 | + |  |
| 152 | China I-A8 | JN936730 | CJS0636D | Jiangsu | Dog | 2006 | + |  |
| 153 | China I-A8 | JN936731 | CJS0639D | Jiangsu | Dog | 2006 | + |  |
| 154 | China I-A8 | JN936732 | CJS0840H | Jiangsu | Human | 2008 | + |  |
| 155 | China I-A8 | JN936733 | CJS0841D | Jiangsu | Dog | 2008 | + |  |
| 156 | China I-A8 | JN936734 | CJS0842D | Jiangsu | Dog | 2008 | + |  |
| 157 | China I-A8 | JN936735 | CJS0843D | Jiangsu | Dog | 2008 | + |  |
| 158 | China I-A8 | JN936736 | CJS0844D | Jiangsu | Dog | 2008 | + |  |
| 159 | China I-A8 | JN936737 | CJS0845D | Jiangsu | Dog | 2008 | + |  |
| 160 | China I-A8 | JN936738 | CJS0846D | Jiangsu | Dog | 2008 | + |  |
| 161 | China I-A8 | JN936739 | CJS0847D | Jiangsu | Dog | 2008 | + |  |
| 162 | China I-A8 | JN936740 | CJS0848D | Jiangsu | Dog | 2008 | + |  |
| 163 | China I-A8 | JN936741 | CJS0849D | Jiangsu | Dog | 2008 | + |  |
| 164 | China I-A8 | JN936742 | CJS0853D | Jiangsu | Dog | 2008 | + |  |
| 165 | China I-A8 | JN936743 | CJS0854D | Jiangsu | Dog | 2008 | + |  |
| 166 | China I-A8 | JN936745 | CJX0902D | Jiangxi | Dog | 2009 | + |  |
| 167 | China I-A8 | JN936748 | CSC0802D | Sichuan | Dog | 2008 | + | ***** |
| 168 | China I-A8 | JN936749 | CSC0803D | Sichuan | Dog | 2008 | + |  |
| 169 | China I-A8 | JN936750 | CSC0805D | Sichuan | Dog | 2008 | + |  |
| 170 | China I-A8 | JN936751 | CSC0807D | Sichuan | Dog | 2008 | + |  |
| 171 | China I-A8 | JN936756 | CSC0912D | Sichuan | Dog | 2009 | + |  |
| 172 | China I-A8 | JN936758 | CSC1014D | Sichuan | Dog | 2010 | + |  |
| 173 | China I-A8 | JN936759 | CSC1015D | Sichuan | Dog | 2010 | + |  |
| 174 | China I-A8 | JN936760 | CSC1016D | Sichuan | Dog | 2010 | + |  |
| 175 | China I-A8 | JN936761 | CSC1017D | Sichuan | Dog | 2010 | + |  |
| 176 | China I-A8 | JN936762 | CSC1018D | Sichuan | Dog | 2010 | + |  |
| 177 | China I-A8 | JN936764 | CSD0614D | Shandong | Dog | 2006 | + |  |
| 178 | China I-A8 | JN936765 | CSD0707D | Shandong | Dog | 2007 | + |  |
| 179 | China I-A8 | JN936766 | CSD0708D | Shandong | Dog | 2007 | + |  |
| 180 | China I-A8 | JN936767 | CSD0710D | Shandong | Dog | 2007 | + |  |
| 181 | China I-A8 | JN936768 | CSD0801D | Shandong | Dog | 2008 | + |  |
| 182 | China I-A8 | JN936769 | CSD0803D | Shandong | Dog | 2008 | + |  |
| 183 | China I-A8 | JN936770 | CSD0804D | Shandong | Dog | 2008 | + |  |
| 184 | China I-A8 | JN936772 | CSD0837D | Shandong | Dog | 2008 | + |  |
| 185 | China I-A8 | JN936773 | CSD0843D | Shandong | Dog | 2008 | + |  |
| 186 | China I-A8 | JN936774 | CSD0932D | Shandong | Dog | 2009 | + |  |
| 187 | China I-A8 | JN936775 | CSD0933D | Shandong | Dog | 2009 | + |  |
| 188 | China I-A8 | JN936777 | CSD0935D | Shandong | Dog | 2009 | + |  |
| 189 | China I-A8 | JN936778 | CSD0936D | Shandong | Dog | 2009 | + |  |
| 190 | China I-A8 | JN936779 | CSD1046H | Shandong | Human | 2010 | + |  |
| 191 | China I-A8 | JN936780 | CSH0408D | Shanghai | Dog | 2004 | + |  |
| 192 | China I-A8 | JN936781 | CSH0410D | Shanghai | Dog | 2004 | + |  |
| 193 | China I-A8 | JN936782 | CSH0501D | Shanghai | Dog | 2005 | + |  |
| 194 | China I-A8 | JN936783 | CSH0503D | Shanghai | Dog | 2005 | + |  |
| 195 | China I-A8 | JN936784 | CSH0505D | Shanghai | Dog | 2005 | + |  |
| 196 | China I-A8 | JN936785 | CSX0901D | Shaanxi | Dog | 2009 | + |  |
| 197 | China I-A8 | JN936786 | CSX0904D | Shaanxi | Dog | 2009 | + |  |
| 198 | China I-A8 | JN936787 | CZJ0803D | Zhejiang | Dog | 2008 | + |  |
| 199 | China I-A8 | JN936789 | CZJ0804D | Zhejiang | Dog | 2008 | + |  |
| 200 | China I-A8 | JN936790 | CZJ0810D | Zhejiang | Dog | 2008 | + |  |
| 201 | China I-A8 | JN936791 | CZJ0811D | Zhejiang | Dog | 2008 | + |  |
| 202 | China I-A8 | JN936792 | CZJ0814D | Zhejiang | Dog | 2008 | + |  |
| 203 | China I-A8 | JN936793 | CZJ0816D | Zhejiang | Dog | 2008 | + |  |
| 204 | China I-A8 | DQ849065 | JSS62 | Jiangsu | Dog | 2005 |  |  |
| 205 | China I-A8 | DQ849066 | JSL26 | Jiangsu | Dog | 2005 |  |  |
| 206 | China I-A8 | DQ849067 | JSL27 | Jiangsu | Dog | 2005 |  |  |
| 207 | China I-A8 | DQ849068 | JSL29 | Jiangsu | Dog | 2005 |  |  |
| 208 | China I-A8 | EF556198 | Zhejiang Wz0(H) | Zhejiang | Human | 2006 |  |  |
| 209 | China I-A8 | EU253477 | Yunnan_Md06 | Yunnan | Dog | 2006 |  |  |
| 210 | China I-A8 | EU267752 | Hebei0(H) | Hebei | Human | 2007 |  | ***** |
| 211 | China I-A8 | EU267773 | Jiangsu Wx1 | Jiangsu | Dog | 2005 |  |  |
| 212 | China I-A8 | EU267775 | Jiangsu Yc37 | Jiangsu | Dog | 2005 |  |  |
| 213 | China I-A8 | EU267776 | Jiangsu Yc58 | Jiangsu | Dog | 2005 |  |  |
| 214 | China I-A8 | EU549783 | BD06 | Hebei | Dog | 2006 |  |  |
| 215 | China I-A8 | EU700029 | BeijingHu1 | Beijing | Human | 2007 |  |  |
| 216 | China I-A8 | EU700030 | Zhejiang Wz1(H) | Zhejiang | Human | 2008 |  |  |
| 217 | China I-A8 | FJ418883 | SBH | Shanghai | Human | 1992 |  |  |
| 218 | China I-A8 | FJ418884 | SBD | Shanghai | Dog | 1992 |  |  |
| 219 | China I-A8 | FJ418886 | SH06 | Shanghai | Dog | 2006 |  |  |
| 220 | China I-A8 | FJ602447 | CQH | Chongqing | Human | 2007 |  |  |
| 221 | China I-A8 | FJ602448 | CXs | Zhejiang | Dog | 2007 |  |  |
| 222 | China I-A8 | FJ602457 | ZJhz | Zhejiang | Dog | 2007 |  |  |
| 223 | China I-A8 | FJ602458 | ZJzj | Zhejiang | Dog | 2007 |  |  |
| 224 | China I-A8 | FJ719758 | ZJQZ | Zhejiang | Dog | 2008 |  |  |
| 225 | China I-A8 | FJ825126 | D03 | Zhejiang | Dog | 2008 |  |  |
| 226 | China I-A8 | FJ825127 | D04 | Zhejiang | Dog | 2008 |  |  |
| 227 | China I-A8 | FJ825128 | D06 | Zhejiang | Dog | 2008 |  |  |
| 228 | China I-A8 | FJ825129 | D08 | Zhejiang | Dog | 2008 |  |  |
| 229 | China I-A8 | FJ825130 | D09 | Zhejiang | Dog | 2008 |  |  |
| 230 | China I-A8 | FJ825131 | D10 | Zhejiang | Dog | 2008 |  |  |
| 231 | China I-A8 | FJ825132 | D11 | Zhejiang | Dog | 2008 |  |  |
| 232 | China I-A8 | FJ825133 | F01 | Zhejiang | Ferret badger | 2008 |  |  |
| 233 | China I-A8 | FJ866832 | FJ010 | Fujian | Dog | 2008 |  |  |
| 234 | China I-A8 | FJ866833 | FJ011 | Fujian | Dog | 2008 |  |  |
| 235 | China I-A8 | FJ866834 | FJ015 | Fujian | Dog | 2008 |  |  |
| 236 | China I-A8 | FJ866835 | FJ008 | Fujian | Dog | 2008 |  |  |
| 237 | China I-A8 | FJ866836 | FJ009 | Fujian | Dog | 2008 |  |  |
| 238 | China I-A8 | GQ857465 | ZJCA1 | Zhejiang | Dog | 2009 |  |  |
| 239 | China I-A8 | GQ857466 | 08ZL01 | Zhejiang | Dog | 2008 |  |  |
| 240 | China I-A8 | GQ857467 | 08ZL02 | Zhejiang | Dog | 2008 |  |  |
| 241 | China I-A8 | GQ857470 | LH | Zhejiang | Dog | 2008 |  |  |
| 242 | China I-A8 | GU186389 | CQJLP01 | Chongqing | Dog | 2008 |  |  |
| 243 | China I-A8 | GU186403 | SDDZ01 | Shandong | Cattle | 2006 |  |  |
| 244 | China I-A8 | GU186404 | SDJN01 | Shandong | Cattle | 2007 |  |  |
| 245 | China I-A8 | GU186405 | SDJN02 | Shandong | Cattle | 2007 |  |  |
| 246 | China I-A8 | GU186409 | TJDB01 | Tianjin | Dog | 2006 |  |  |
| 247 | China I-A8 | GU186410 | TJDB02 | Tianjin | Dog | 2007 |  |  |
| 248 | China I-A8 | GU186411 | TJDB03 | Tianjin | Dog | 2008 |  |  |
| 249 | China I-A8 | GU591789 | Shaanxi-HZ-6 | Shaanxi | Dog | 2009 |  |  |
| 250 | China I-A8 | GU591791 | Sichuan-BZ-1 | Sichuan | Dog | 2009 |  |  |
| 251 | China I-B | JN936721 | CHN0901D | Hunan | Dog | 2009 | + | ***** |
| 252 | China I-B | JN936722 | CHN0902D | Hunan | Dog | 2009 | + |  |
| 253 | China I-B | JN936723 | CHN0903D | Hunan | Dog | 2009 | + | ***** |
| 254 | China I-B | JN936724 | CHN0906H | Hunan | Human | 2009 | + | ***** |
| 255 | China II-A | JN936788 | CZJ0803F | Zhejiang | Ferret badger | 2008 | + | ***** |
| 256 | China II-A | FJ719749 | JX08-47 | Jiangxi | Ferret badger | 2008 |  | ***** |
| 257 | China II-A | FJ719752 | JX08-48 | Jiangxi | Ferret badger | 2008 |  |  |
| 258 | China II-A | FJ719756 | ZJ-LA | Zhejiang | Ferret badger | 2008 |  |  |
| 259 | China II-A | FJ825134 | F03 | Zhejiang | Ferret badger | 2008 |  |  |
| 260 | China II-A | FJ825135 | F05 | Zhejiang | Ferret badger | 2008 |  |  |
| 261 | China II-A | GQ857468 | 08ZL11 | Zhejiang | Ferret badger | 2008 |  |  |
| 262 | China II-A | GQ857469 | 08ZL13 | Zhejiang | Ferret badger | 2008 |  |  |
| 263 | China II-B | JN936688 | CGD0801D | Guangdong | Dog | 2008 | + | ***** |
| 264 | China II-B | JN936689 | CGX0511D | Guangxi | Dog | 2005 | + |  |
| 265 | China II-B | JN936690 | CGX0601D | Guangxi | Dog | 2006 | + |  |
| 266 | China II-B | JN936693 | CGX0614D | Guangxi | Dog | 2006 | + |  |
| 267 | China II-B | JN936695 | CGX0801D | Guangxi | Dog | 2008 | + | ***** |
| 268 | China II-B | JN936697 | CGZ0508D | Guizhou | Dog | 2005 | + |  |
| 269 | China II-B | JN936707 | CHN0527D | Hunan | Dog | 2005 | + |  |
| 270 | China II-B | JN936711 | CHN0633D | Hunan | Dog | 2006 | + |  |
| 271 | China II-B | JN936713 | CHN0642D | Hunan | Dog | 2006 | + |  |
| 272 | China II-B | JN936715 | CHN0803D | Hunan | Dog | 2008 | + |  |
| 273 | China II-B | JN936719 | CHN0812D | Hunan | Dog | 2008 | + |  |
| 274 | China II-B | JN936720 | CHN0813H | Hunan | Human | 2008 | + | ***** |
| 275 | China II-B | DQ849071 | GX4 | Guangxi | Dog | 1994 |  |  |
| 276 | China II-B | EU267746 | Guizhou Al01 | Guizhou | Dog | 2005 |  |  |
| 277 | China II-B | EU267747 | Guizhou Al03 | Guizhou | Dog | 2005 |  | ***** |
| 278 | China II-B | FJ418880 | GX2 | Guangxi | Dog | 1994 |  |  |
| 279 | China II-B | FJ602452 | LU | Henan | Deer | 1993 |  |  |
| 280 | China II-B | GQ472535 | GX01 | Guangxi | Dog | 2004 |  |  |
| 281 | China II-B | GQ472536 | GX08 | Guangxi | Dog | 2003 |  |  |
| 282 | China II-B | GQ472537 | GX09 | Guangxi | Dog | 2003 |  |  |
| 283 | China II-B | GQ472538 | GX014 | Guangxi | Dog | 2003 |  |  |
| 284 | China II-B | GQ472540 | GX091 | Guangxi | Dog | 2004 |  |  |
| 285 | China II-B | GQ472541 | GX195 | Guangxi | Dog | 2004 |  |  |
| 286 | China II-B | GQ472543 | GX260 | Guangxi | Dog | 2004 |  |  |
| 287 | China II-B | GQ472546 | GXHX | Guangxi | Dog | 2005 |  |  |
| 288 | China II-B | GQ472547 | GXHXB | Guangxi | Dog | 2007 |  |  |
| 289 | China II-B | GQ472549 | GXLA | Guangxi | Dog | 2003 |  |  |
| 290 | China II-B | GQ472550 | GXLB | Guangxi | Dog | 2007 |  |  |
| 291 | China II-B | GQ472554 | GXNND | Guangxi | Dog | 2007 |  |  |
| 292 | China II-B | GQ472557 | GXQZD | Guangxi | Dog | 2006 |  |  |
| 293 | China II-B | GQ472558 | GXSL | Guangxi | Cattle | 2005 |  |  |
| 294 | China II-B | GQ472559 | GXWX | Guangxi | Pig | 2005 |  |  |
| 295 | China II-B | GU186379 | HuNDB02 | Hunan | Dog | 2005 |  | ***** |
| 296 | China II-B | GU186380 | HuNDB03 | Hunan | Dog | 2006 |  |  |
| 297 | China II-B | GU186386 | HuNPB01 | Hunan | Pig | 2006 |  |  |
| 298 | China II-B | GU186398 | GDMM55 | Guangdong | Dog | 2007 |  |  |
| 299 | China II-B | GU186399 | GDMM57 | Guangdong | Dog | 2007 |  |  |
| 300 | China II-B | GU186400 | GDZQ45 | Guangdong | Dog | 2007 |  |  |
| 301 | China II-B | GU186401 | GDZQ46 | Guangdong | Dog | 2007 |  |  |
| 302 | China II-B | GU186402 | GXLA01 | Guangxi | Dog | 2003 |  |  |
| 303 | China II-B | L04523 | CGX89-1 | Guangxi | Dog | 1993 |  |  |
| 304 | China III-A | DQ875050 | MRV | Henan | Mouse | 1989 |  | ***** |
| 305 | China III-B | EU267745 | Guizhou Al0(H) | Guizhou | Human | 2004 |  |  |
| 306 | China III-B | EU267772 | Jiangsu Wx0(H) | Jiangsu | Human | 2004 |  | ***** |
| 307 | ChinaI V | EU284095 | NeiMeng927A | Neimenggu | Raccoon dog | 2007 |  |  |
| 308 | China IV | EU284096 | NeiMeng927B | Neimenggu | Raccoon dog | 2007 |  | ***** |
| 309 | China IV | EU284097 | NeiMeng1025B | Neimenggu | Raccoon dog | 2007 |  |  |
| 310 | China IV | EU284098 | NeiMeng1025C | Neimenggu | Raccoon dog | 2007 |  |  |
| 311 | China V | AY009098 | CNX8511 | Ningxia | Human | 1985 |  |  |
| 312 | China V | AY009099 | CNX8601 | Ningxia | Human | 1986 |  |  |
| 313 | China V | DQ849072 | CQ92 | Chongqing | Dog | 1992 |  | ***** |
| 314 | China V | GU186387 | CQFJ01 | Chongqing | Dog | 2005 |  | ***** |
| 315 | China V | GU186396 | CQWL02 | Chongqing | Dog | 2005 |  | ***** |
| 316 | China V | GU186397 | CQWS01 | Chongqing | Dog | 2005 |  | ***** |
| 317 | China VI-A | JQ730682 | CYN1009D | Yunnan | Dog | 2010 | + | ***** |
| 318 | China VI-A | EU275242 | Yunnan_Tc06 | Yunnan | Dog | 2006 |  | ***** |
| 319 | China VI-B | DQ849069 | N11 | Guangxi | Dog | 1997 |  | ***** |
| 320 | China VI-B | GQ472552 | GXN119 | Guangxi | Dog | 2000 |  | ***** |

New sequences collected as part of the surveillance program are marked with a '+' in the rightmost column.
