# Supplementary material for: Molecular Phylodynamic Analysis Indicates Lineage Displacement Occurred in Chinese Rabies Epidemics between 1949 to 2010
Source: PLoS Negl Trop Dis. 2013 Jul 11;7(7):e2294. doi: 10.1371/journal.pntd.0002294 (PMC3708843; doi:10.1371/journal.pntd.0002294)
Supplement: Table S2 — Background information of extra sequences used in the World tree ( Figure 4 ). (DOC) [file pntd.0002294.s003.doc]

**Supplementary Table S2:** Background information G sequences from outside China used for world tree in figure 3b.

| **Genbank No.** | **Strain** | **Country** | **Host** | **Year** |
| --- | --- | --- | --- | --- |
| AB110657 | BRhm6 | Argentina | Human | 1999 |
| AB115921 | SN01-23 | Indonesia | Dog | 2001 |
| AB373640 | BR-BAT16 | Cote d'Ivoire | Bat | 2006 |
| AB569299 | H-08-1320 | Sri Lanka | Human | 2008 |
| AF233275 | PV11 | India | Sheep | 1995 |
| AF325464 | POL1-RD | Brazil | Raccoon dog | 1985 |
| AF325472 | 88011 | Iran | Human | 1988 |
| AF325473 | USA3-SK | Canada | Skunk | 1981 |
| AF325477 | MEX1-DG | Canada | Dog | 1991 |
| AF325479 | NGA1-HM | Canada | Human | 1983 |
| AF325487 | 86077 | Malaysia | Human | 1985 |
| AF325489 | V120 | Nepal | Dog | 1989 |
| AF325492 | MEX2-VP | Canada | Bat | 1987 |
| AF325494 | USA8-BT | Canada | Bat | 1981 |
| AF401287 | 9107MAR | Canada | Human | 1990 |
| AY956319 | RABV | India | Human | 2005 |
| DQ076094 | SKRRD0204CW | South Korea | Raccoon dog | 2002 |
| DQ076099 | SKRRD9903YG | South Korea | Raccoon dog | 1999 |
| DQ420624 | 04033MAD | Cote d'Ivoire | human | 2004 |
| EF151231 | BLY 1/99 | India | Dog | 1999 |
| EU086128 | 04029AFG | Afghanistan | Dog | 2004 |
| EU086129 | 9913BIR | Myanmar | Dog | 1999 |
| EU086130 | 9808CBG | Cambodia | Dog | 1999 |
| EU086151 | 03003INDO | Indonesia | Dog | 2003 |
| EU086152 | 9910LAO | Laos | Dog | 1999 |
| EU086153 | 9901NEP | Nepal | Dog | 1998 |
| EU086155 | 04030PHI | Philippines | Human | 2004 |
| EU086156 | 94257SRI | Sri Lanka | Dog | 1996 |
| EU086160 | 01017VNM | Viet Nam | Dog | 2001 |
| EU293115 | 9147FRA | USA | Fox | 1991 |
| EU293116 | 9704ARG | USA | Bat | 1997 |
| GQ233040 | GQ233040 | India | Dog | 2001 |
| GQ303557 | UA341 | Thailand | Human | 2009 |
| GU937024 | KRVR0906 | South Korea | Raccoon dog | 2009 |
| HQ232301 | D713d | Thailand | Dog | 2002 |
| U11737 | 93RABN0113 | Canada | Arctic fox | 1993 |
| U11755 | 91RABN1578 | CAR | Red fox | 1991 |
