# Supplementary material for: Molecular Phylodynamic Analysis Indicates Lineage Displacement Occurred in Chinese Rabies Epidemics between 1949 to 2010
Source: PLoS Negl Trop Dis. 2013 Jul 11;7(7):e2294. doi: 10.1371/journal.pntd.0002294 (PMC3708843; doi:10.1371/journal.pntd.0002294)
Supplement: Table S3 — The Pearson's Chi-squared test results for the differences in geographical composition. (DOC) [file pntd.0002294.s004.doc]

**Supplementary Table S3**

**Statistical Analysis of Geographical Composition of branches of Subclade China I-A**

Provinces with recorded human cases were classified as high, medium or low incidence regions as described in Materials and Methods and are summarized in Table 1. For clade I-A, by summing over provinces in Table 1 according to this classification, we obtain the following table

:

Table S1.1: Number of cases by incidence region and branches for subclade I-A.

|  | **A1** | **A2** | **A3** | **A4** | **A5** | **A6** | **A7** | **A8** |
| --- | --- | --- | --- | --- | --- | --- | --- | --- |
| **High** | 1 | 4 | 20 | 1 | 27 | 0 | 6 | 1 |
| **Middle** | 7 | 55 | 3 | 1 | 5 | 10 | 1 | 90 |
| **Low** | 0 | 0 | 0 | 3 | 0 | 0 | 0 | 15 |

Many of the cells contain insufficient data for the Pearson's chi-squared test and, since we are primarily interested in comparing the geographical composition of branchA8 (the youngest clade which is responsible for the majority of cases in the last three to four years) to the remaining A branches, we merge A1 to A7 into a single sum.

Table S1.2: Number of cases by incidence region and branches for branch I-A8 and . branches I-A1 to A7

|  | **A1-7** | **A8** |
| --- | --- | --- |
| High | **59** | **1** |
| Middle | **82** | **90** |
| Low | **3** | **15** |

We can now perform a Pearson's Chi-squared test on this merged contingency table (Table S1.2) between branches I-A1-7 and branch I-A8. The result shows that an extremely significant difference exists between the proportions (high, middle and low) of two clades groups (P = 9.126×10^-14^. This strongly suggests that the geographic distribution of cases in branch I-A8 is absolutely distinct from the distribution in branches I-A1 to A7.

To further explore the differences between branch I-A8 and branches I-A1 to A7 for high and middle regions and middle and low regions, we proceeded to perform Pearson's Chi-squared test on the decomposed tables (Table S1.3 and Table S1.4). Again, we determined there is a significant difference between the proportions of middle and low incidence events for I-A8 and I-A1 to A7 (P = 0.02331 - Table S1.3), and an extremely significant difference between the proportions of high and middle incidence events for I-A8 and I-A1 to A7 (1.319×10^-11^ - Table S1.4).

Table S1.3: Two way contingency table for Middle and Low Incidence regions and branch I-A8 and branches I-A1 to A7

|  | **A1-7** | **A8** |
| --- | --- | --- |
| Middle | **82** | **90** |
| Low | **3** | **15** |

Significant difference exists between the proportions of high and middle incidence events for branches I-A8 and I-A1 to A7 (P = 0.02331)

Table S1.4: Two way contingency table for high and middle Incidence regions and branch I-A8 and branches I-A1 to A7

|  | **A1-7** | **A8** |
| --- | --- | --- |
| High | **59** | **1** |
| Middle | **82** | **90** |

Extremely significant difference exists between the proportions of high and middle incidence events for I-A8 and I-A1 to A7P value (P = 1.319×10^-11^)

Details of analysis

Hypotheses

**Table S1.2:**

H_0_: the proportions of high, middle and low are independent of columns

H_A_: the proportions of high, middle and low are dependent of columns

Pearson's Chi-squared test

data: x

X-squared = 60.0502, df = 2, p-value = 9.126e-14

from the above highlighted result, at level 0.05, we reject the null hypothesis and accept the alternative hypothesis instead.

**Table S1.3:**

H_0_: the proportions of high and middle events are independent of columns

H_A_: the proportions of high and middle events are dependent of columns

> chisq.test(x1)

Pearson's Chi-squared test with Yates' continuity correction

data: x1

X-squared = 45.7857, df = 1, p-value = 1.319e-11

at level 0.05, we reject the null hypothesis and accept the alternative hypothesis instead.

**Table S1.4:**

H_0_: the proportions of middle and low events are independent of columns

H_A_: the proportions of middle and low events are dependent of columns

> chisq.test(x2)

Pearson's Chi-squared test with Yates' continuity correction

data: x2

X-squared = 5.1449, df = 1, p-value = 0.02331

at level 0.05, we reject the null hypothesis and accept the alternative hypothesis instead.
