# Supplementary material for: Molecular Phylodynamic Analysis Indicates Lineage Displacement Occurred in Chinese Rabies Epidemics between 1949 to 2010
Source: PLoS Negl Trop Dis. 2013 Jul 11;7(7):e2294. doi: 10.1371/journal.pntd.0002294 (PMC3708843; doi:10.1371/journal.pntd.0002294)
Supplement: Table S4 — Background information of wildlife sequences collected from N and G sequences from this study and previously submitted to GenBank. (DOC) [file pntd.0002294.s005.doc]

|  | **CLADE** | **STRAIN** | **PROVINCE** | **HOST** | **YEAR** | **ACCESSION NUMBER** | |
| --- | --- | --- | --- | --- | --- | --- | --- |
|  |  |  |  |  |  | **N** | **G** |
| 1 | I | JX09-17(fb) | Jiangxi | ferret badger | 2009 | JU233765 | GU233763 |
| 2 | I | F01 | Zhejiang | ferret badger | 2008 | FJ032318 | FJ825133 |
| 3 | II | 08ZL11 | Zhejiang | ferret badger | 2008 |  | GQ857468 |
| 4 | II | 08ZL13 | Zhejiang | ferret badger | 2008 |  | GQ857469 |
| 5 | II | F02 | Zhejiang | ferret badger | 2008 | FJ712195 | FJ712195 |
| 6 | II | F03 | Zhejiang | ferret badger | 2008 |  | FJ825134 |
| 7 | II | F04 | Zhejiang | ferret badger | 2008 | FJ712196 | FJ712196 |
| 8 | II | F05 | Zhejiang | ferret badger | 2008 |  | FJ825135 |
| 9 | II | JX08-45 | Jiangxi | ferret badger | 2008 | GU647092 | GU647092 |
| 10 | II | JX08-47 | Jiangxi | ferret badger | 2008 | FJ719751 | FJ719749 |
| 11 | II | JX08-48 | Jiangxi | ferret badger | 2008 | FJ719753 | FJ719752 |
| 12 | II | ZJ-LA | Zhejiang | ferret badger | 2008 | FJ598135 | FJ719756 |
| 13 | II | 02049CHI | Henan | sika deer | 1993 | EU086184 |  |
| 14 | II | JX08-58 | Jiangxi | ferret badger | 2008 | FJ719755 |  |
| 15 | II | CZJ0803F | Zhejiang | ferret badger | 2008 | JN974877 |  |
| 16 | II | ZJF1 | Zhejiang | ferret badger | 2008 | HQ118114 |  |
| 17 | II | XSN5 | Zhejiang | mouse | 2008 | JQ799140 |  |
| 18 | II | JX12-102 | Jiangxi | ferret badger | 2012 | JQ950450 | JQ950451 |
| 19 | II | JX12-234 | Jiangxi | ferret badger | 2012 | JQ950452 | JQ950453 |
| 20 | II | JX12-67 | Jiangxi | ferret badger | 2012 | JQ950448 | JQ950449 |
| 21 | II | JX12-64 | Jiangxi | ferret badger | 2012 |  | JQ950447 |
| 22 | II | CHpg2 | Guangxi | wild pig | 2005 | AB294349 |  |
| 23 | II | ZJF2 | Zhejiang | ferret badger | 2008 | HQ118115 |  |
| 24 | II | ZJF3 | Zhejiang | ferret badger | 2008 | HQ118116 |  |
| 25 | II | ZJF4 | Zhejiang | ferret badger | 2008 | HQ118117 |  |
| 26 | II | ZJF5 | Zhejiang | ferret badger | 2008 | HQ118118 |  |
| 27 | III | DRV | Jilin | deer | 1989 | DQ875051 | DQ875051 |
| 28 | III | LU | Henan | deer | 1993 | EU159378* | FJ602452 |
| 29 | III | MRV | Henan | mouse | 1989 | DQ875050 | DQ875050 |
| 30 | III | CHVC06 | Yunnan | mouse | 2006 | EU282381 |  |
| 31 | IV | NeiMeng1025B | Inner mongolia | raccoon dog | 2007 | EU652445 | EU284097 |
| 32 | IV | NeiMeng1025C | Inner mongolia | raccoon dog | 2007 | EU284094 | EU284098 |
| 33 | IV | NeiMeng925 | Inner mongolia | raccoon dog | 2008 | FJ415313 | FJ415313 |
| 34 | IV | NeiMeng927A | Inner mongolia | raccoon dog | 2007 | EU284093 | EU284095 |
| 35 | IV | NeiMeng927B | Inner mongolia | raccoon dog | 2007 | EU652444 | EU284096 |

**Supplementary Table S4:** Wildlife isolates collected in China between 1993 and 2012. Glycoprotein (G) sequences are from table S1. Nucleoprotein (N) sequences are from taken from dataset in Guo 2013.
