# Supplementary material for: Molecular Phylodynamic Analysis Indicates Lineage Displacement Occurred in Chinese Rabies Epidemics between 1949 to 2010
Source: PLoS Negl Trop Dis. 2013 Jul 11;7(7):e2294. doi: 10.1371/journal.pntd.0002294 (PMC3708843; doi:10.1371/journal.pntd.0002294)
Supplement: Table S5 — Comparison of dog and wildlife composition of Chinese rabies lineages. (DOC) [file pntd.0002294.s006.doc]

**Table S5. Comparison of dog and wildlife composition of Chinese rabies lineages**

To investigate the composition of wildlife and domestic dog isolates amongst the six China lineages (China I, China II, China III (Cosmopolitan), China IV(Arctic), China V and China VI), we combined all domestic dog and wildlife isolates in the glycoprotein sequence dataset from this study with domestic dog and wildlife isolates in the nucleoprotein sequence from our earlier study. Duplicate N & G entries were combined and China V & VI had insufficient numbers of sequences for statistical analyses and were removed, leaving a final dataset of 368 isolates (Table S3.1).

**Table S5**.1 Total number of domestic dog and wildlife isolates in combined N and G dataset.

| **Clade** | **Wild strains** | **Dog strains** |
| --- | --- | --- |
| **China I** | 2 | 272 |
| **China II** | 15 | 60 |
| **China III** | 3 | 11 |
| **China IV** | 5 | 0 |

We conducted the Fisher’s exact test on the decomposed tables (Table S5.2, Table S5.3, Table S5.4, and Table S5.5).

**Table S5**.2. China I vs China II decomposed table

| **Clade** | **Wild strains** | **Dog strains** |
| --- | --- | --- |
| **China I** | 2 | 272 |
| **China II** | 15 | 60 |

**Table S5.3. China II vs China III** decomposed table

| **Clade** | **Wild strains** | **Dog strains** |
| --- | --- | --- |
| **China II** | 15 | 60 |
| **China III** | 3 | 11 |

**Table S5.4. China II vs China IV decomposed table**

| **Clade** | **Wild strains** | **Dog strains** |
| --- | --- | --- |
| **China II** | 15 | 60 |
| **China IV** | 5 | 0 |

**Table S5.5. China III vs China IV decomposed table**

| **Clade** | **Wild strains** | **Dog strains** |
| --- | --- | --- |
| **China III** | 3 | 11 |
| **China IV** | 5 | 0 |

We identified extremely significant differences between the proportions of wild strains and dog strains for China I and II as well as for China II and China IV (P value= 2.74×10^-09^ and P value = 0.00064 respectively), and very significant difference between the proportions of wild strains and dog strains for China III and IV (P value = 0.0048), whereas no significant difference between the proportions of wild strains and dog strains for China II and III (P value = 1) .
