# Supplementary material for: Molecular Phylodynamic Analysis Indicates Lineage Displacement Occurred in Chinese Rabies Epidemics between 1949 to 2010
Source: PLoS Negl Trop Dis. 2013 Jul 11;7(7):e2294. doi: 10.1371/journal.pntd.0002294 (PMC3708843; doi:10.1371/journal.pntd.0002294)

**Supplementary Table S6. Human rabies cases in China by province and year.**  The numbers of human cases in each province or municipality of China from 1996 to 2010 for each province or municipality. Provinces are grouped and colored according to high, medium, low and very low incidence regions defined in Figure 2


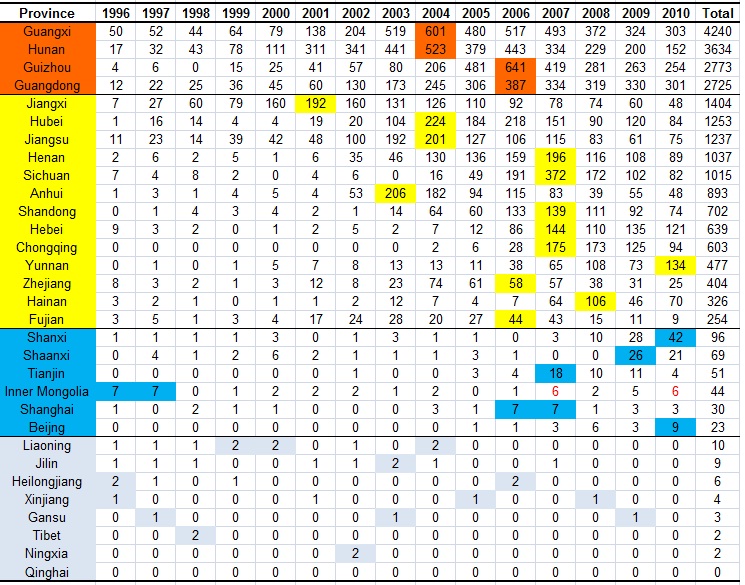

Supplement: Table S6 — The numbers of human cases in each province or municipality of China from 1996 to 2010. (DOC) [file pntd.0002294.s007.doc]
