# Supplementary material for: Molecular Phylodynamic Analysis Indicates Lineage Displacement Occurred in Chinese Rabies Epidemics between 1949 to 2010
Source: PLoS Negl Trop Dis. 2013 Jul 11;7(7):e2294. doi: 10.1371/journal.pntd.0002294 (PMC3708843; doi:10.1371/journal.pntd.0002294)
Supplement: Table S7 — Number of collected isolates versus number of human rabies cases by province. (DOC) [file pntd.0002294.s008.doc]

**Supplementary Table S7:** Number of collected isolates versus number of human rabies cases by province.

| **Province** | **No of isolates** | **No of human cases** | **Population (x10,000)** | **Population**  **Density (/m^2^)** | **Incidence** |
| --- | --- | --- | --- | --- | --- |
| **Anhui** | 37 | 893 | 5968 | 426.29 | M |
| **Beijing** | 1 | 23 | 2019 | 1201.21 | L |
| **Chongqing** | 13 | 603 | 2919 | 354.68 | M |
| **Fujian** | 5 | 254 | 3720 | 305.67 | M |
| **Gansu** | 0 | 3 | 2564 | 56.48 | V |
| **Guangdong** | 5 | 2725 | 10505 | 590.17 | H |
| **Guangxi** | 44 | 4240 | 4645 | 196.27 | H |
| **Guizhou** | 18 | 2773 | 3469 | 199.37 | H |
| **Hainan** | 0 | 326 | 877 | 258.40 | M |
| **Hebei** | 2 | 639 | 7241 | 385.78 | M |
| **Heilongjiang** | 0 | 6 | 3834 | 81.75 | V |
| **Henan** | 10 | 1037 | 9388 | 562.16 | M |
| **Hubei** | 10 | 1253 | 5758 | 309.74 | M |
| **Hunan** | 40 | 3634 | 6596 | 311.13 | H |
| **Jiangsu** | 28 | 1237 | 7899 | 769.88 | M |
| **Jiangxi** | 8 | 1404 | 4488 | 269.39 | M |
| **Jilin** | 0 | 9 | 2749 | 146.69 | V |
| **Liaoning** | 0 | 10 | 4383 | 300.21 | V |
| **Neimenggu** | 0 | 44 | 2482 | 20.68 | L |
| **Ningxia** | 2 | 2 | 639 | 96.23 | V |
| **Qinghai** | 0 | 0 | 568 | 7.89 | V |
| **Shaanxi** | 3 | 69 | 3743 | 182.05 | L |
| **Shandong** | 19 | 702 | 9637 | 615.00 | M |
| **Shanghai** | 8 | 30 | 2347 | 3701.89 | L |
| **Shanxi** | 3 | 96 | 3593 | 230.32 | L |
| **Sichuan** | 17 | 1015 | 8050 | 165.06 | M |
| **Tianjin** | 3 | 51 | 1355 | 1198.80 | L |
| **Tibet** | 0 | 2 | 303 | 2.46 | V |
| **Xinjiang** | 0 | 4 | 2209 | 13.81 | V |
| **Yunnan** | 9 | 477 | 4631 | 117.54 | M |
| **Zhejiang** | 31 | 404 | 5463 | 536.64 | M |
